# Proteomics: A Tool to Study Platelet Function

**DOI:** 10.3390/ijms22094776

**Published:** 2021-04-30

**Authors:** Olga Shevchuk, Antonija Jurak Begonja, Stepan Gambaryan, Matthias Totzeck, Tienush Rassaf, Tobias B. Huber, Andreas Greinacher, Thomas Renne, Albert Sickmann

**Affiliations:** 1 Leibniz-Institut für Analytische Wissenschaften—ISAS—e.V, Bunsen-Kirchhoff-Straße 11, 44139 Dortmund, Germany; 2 Department of Immunodynamics, Institute of Experimental Immunology and Imaging, University Hospital Essen, Hufelandstrasse 55, 45147 Essen, Germany; 3 Department of Biotechnology, University of Rijeka, Radmile Matejčić 2, 51000 Rijeka, Croatia; ajbegonja@biotech.uniri.hr; 4 Sechenov Institute of Evolutionary Physiology and Biochemistry, Russian Academy of Sciences, Torez pr. 44, 194223 St. Petersburg, Russia; s.gambaryan@klin-biochem.uni-wuerzburg.de; 5 West German Heart and Vascular Center, Department of Cardiology and Vascular Medicine, University Hospital Essen, Hufelandstrasse 55, 45147 Essen, Germany; Matthias.totzeck@uk-essen.de (M.T.); tienush.rassaf@uk-essen.de (T.R.); 6 III. Department of Medicine, University Medical Center Hamburg-Eppendorf, 20246 Hamburg, Germany; t.huber@uke.de; 7 Institut für Immunologie und Transfusionsmedizin, Universitätsmedizin Greifswald, Sauerbruchstraße, 17475 Greifswald, Germany; andreas.greinacher@med.uni-greifswald.de; 8 Institute of Clinical Chemistry and Laboratory Medicine, University Medical Center Hamburg-Eppendorf, Martinistrasse 52, 20246 Hamburg, Germany; thomas@renne.net; 9 Medizinisches Proteom-Center (MPC), Medizinische Fakultät, Ruhr-Universität Bochum, 44801 Bochum, Germany; 10 Department of Chemistry, College of Physical Sciences, University of Aberdeen, Aberdeen AB24 3FX, UK

**Keywords:** platelets, signaling, mass spectrometry, LC-MS/MS, PTMs, phosphoproteomics, targeted proteomics, platelet transfusion proteomics, precision medicine

## Abstract

Platelets are components of the blood that are highly reactive, and they quickly respond to multiple physiological and pathophysiological processes. In the last decade, it became clear that platelets are the key components of circulation, linking hemostasis, innate, and acquired immunity. Protein composition, localization, and activity are crucial for platelet function and regulation. The current state of mass spectrometry-based proteomics has tremendous potential to identify and quantify thousands of proteins from a minimal amount of material, unravel multiple post-translational modifications, and monitor platelet activity during drug treatments. This review focuses on the role of proteomics in understanding the molecular basics of the classical and newly emerging functions of platelets. including the recently described role of platelets in immunology and the development of COVID-19.The state-of-the-art proteomic technologies and their application in studying platelet biogenesis, signaling, and storage are described, and the potential of newly appeared trapped ion mobility spectrometry (TIMS) is highlighted. Additionally, implementing proteomic methods in platelet transfusion medicine, and as a diagnostic and prognostic tool, is discussed.

## 1. Platelets Biology and Functions

### 1.1. The Platelet’s Origin

Platelets are small, anucleated cell fragments, and besides erythrocytes, they are the second most abundant in blood circulation. They are derived from the cytoplasm of megakaryocytes (MK), the large cells in the bone marrow and lungs. They circulate in the bloodstream for 7 to 10 days, and are cleared in the spleen or liver after they become senile [1,2,3]. The platelet production process—thrombopoiesis—is spatially regulated by the bone marrow vasculature and stimulated by thrombopoietin, a glycoprotein hormone primarily produced in the liver parenchymal cells and kidney [4]. Thrombopoietin binding to myeloproliferative leukemia protein (c-Mpl or CD110) predominantly expressed on megakaryocyte surface induces the receptor’s homodimerization, and activates JAK-STAT and MAPK signaling cascades that subsequently control cellular proliferation and differentiation to platelets [5,6]. Additionally, it was described to induce changes in the mitochondrial metabolism of hematopoietic stem cells and prime them to undergo direct differentiation to megakaryocytes [7]. Platelet-derived extracellular vesicles from platelets activated during inflammation have been shown to infiltrate the bone marrow and activate MKs and MK-precursor cells for rapid reconstitution of a number of circulating platelets [8].

### 1.2. Platelets in Hemostasis

**Figure 1:**
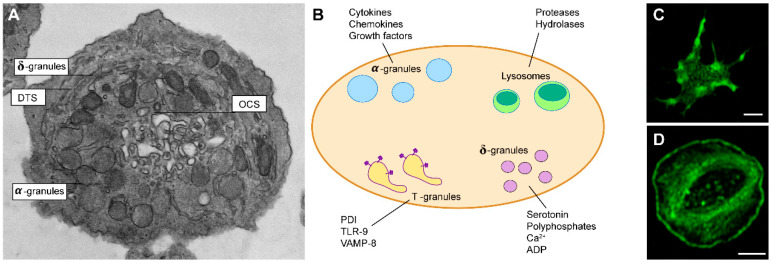
(**A**) Electron micrograph of resting platelet. Indicated: open canalicular system (OCS), dense tubular system (DTS), α-granules, δ-granules that contain small molecules. (**B**) Graphical representation of the main secretory granules of platelets and their contents. Indicated: protein disulfide isomerase (PDI), with toll-like receptor 9 (TLR), vesicle-associated membrane protein 8 (VAMP-8). (**C**) A platelet forming filopodia and (**D**) a fully spread platelet. Platelets were stained for F-actin with phalloidin-Alexa Fluor 488, and visualized by a confocal laser scanning microscope.

The first described and main role of platelets is hemostasis (from “hemo”—Greek blood, “stasis”—stable) or prevention of blood loss. The primary steps of hemostasis are performed by platelets, the secondary by proteases of the coagulation cascade. Fibrinolysis is an additional process of dissolving the created clots, restoring the normal blood circulation, and bringing the whole system to initial equilibrium. Platelet activation is central for the execution of platelets’ diverse functions from primary hemostasis to inflammation. Such activation is mediated by numerous receptors present on their surface and bioactive molecules stored in different granules within platelets (Figure 1A,B). Upon platelet activation, the cargo from different types of granules and microparticles is released in a well-regulated manner, and the resulting specific platelet “releasates” create a microenvironment of biologically active metabolites and proteins. The specific content of “releasates” is crucial during platelet aggregation and thrombus formation, due to the efficient delivery of growth factors and immune modulators to their sites of effect, and the enhancement of the coagulative response in a positive feedback loop [8]. Three types of granules can be recognized within platelets that harbor molecules essential for their function: α-granules that contain diverse proteins, cytokines, chemokines, and growth factors; δ-granules that contain small molecules-serotonin, ADP, polyphosphates, calcium; and lysosomes that contain degrading enzymes [9]. The content of α-granules is secreted through special surface-connected channels called the open canalicular system (OCS). It facilitates membrane remodeling and shape changes during platelet adhesion, including filopodia formation and irreversible spreading [10,11]. The platelet dense tubular system (DTS) is central in the initiation and modulation of platelet activation. The platelet prostaglandin and thromboxane synthesis and an internal calcium store critical to platelet activation are both found in the platelet DTS [12]. Visualization of platelet thin frozen sections shows DTS membranes as small vesicular structures termed T granules [11]. T-granules contain toll-like receptor 9 (TLR9), protein disulfide isomerase (PDI), and VAMP-8, and it has been suggested that these are recruited to the cell surface contributing to the secretion [11,13].

Platelets are activated when exposed to extracellular matrix proteins in the areas of endothelial damage. Platelets surveil the endothelium surface within the blood vessels and are especially important to stop bleedings in the small capillaries. Endothelial cells contribute to keeping them inactivated by secreting prostaglandin I2 (prostacyclin, PGI2) and nitric oxide (NO), as well as expressing CD39 (ectonucleotidase that cleaves ADP/ATP). Upon vessel wall injury, platelets quickly recognize the damage; they adhere to the damaged areas and are activated to seal the injury [14]. Platelet adhesion is initiated by detecting extracellular matrix proteins, such as collagen or fibronectin, which are exposed to the circulating blood upon endothelial injury. The platelets’ binding mechanisms depend on the rate of shear stress that differ in arterial and venous systems. Under high shear conditions, the plasma-derived von Willebrand factor (VWF) changes its conformation and binds to the exposed collagen at the damaged site [14,15]. Under the high shear conditions, the GPIb-V-IX receptor complex is required to stabilize platelet adhesion to the vessel surface, besides collagen receptor glycoprotein (GP) VI and αIIbβ3 integrin, which indirectly interact with collagen via VWF [16]. Recent proteomics studies describe both the positive and negative influences of N-glycosylation on collagen-platelets interactions [17]. At low shear conditions, the binding to collagen via α2β1 integrin has a major role in platelet adhesion to the injured endothelium.

Initial engagement of receptors leads to the activation of platelets through multiple signaling pathways that precede cytoskeletal rearrangements. Upon activation, platelets change their shape from discoid to more spherical, form filopodia, and fully spread into fried-egg shape lamellipodia (Figure 1C,D). Following adhesion, activated platelets act in a paracrine and autocrine way to recruit additional platelets from circulation and to further activate themselves. This process is mediated via the production of thromboxane A2 (TxA2) and the release of ADP from δ-granules, stimulating TxA2 and P2Y (1 and 12) receptors. In this way, additional platelets will adhere to the site of the injury, aggregate, and build up three-dimensional clots with the tightly regulated hierarchical architecture of activated platelets, coagulation factors, regulatory factors, and fibrin [18].

The release of α-granules from activated platelets increases platelet surface expression of P-selectin and CD40L that enables them to interact with circulating leukocytes and endothelium. An increased level of circulating monocyte–platelet aggregates (MPAs) represents one of the most robust platelet activation markers [19]. Upon platelet activation, integrin αIIbβ3 adopts open conformation and binds to the fibrinogen and VWF. This binding enables interconnecting platelets to each other and additionally stabilizing aggregates. Finally, exposed damaged tissue releases tissue factor that will stimulate thrombin formation. Thrombin cleaves and stimulates PAR (protease-activated receptor, in humans 1 and 4; in mice 3 and 4) on platelets and cleaves fibrinogen into fibrin that will further strengthen aggregates [20]. The clot’s final stabilization is mediated via actin–myosin platelet retraction as part of secondary hemostasis.

### 1.3. Platelets Activation and Inhibition

**Figure 2:**
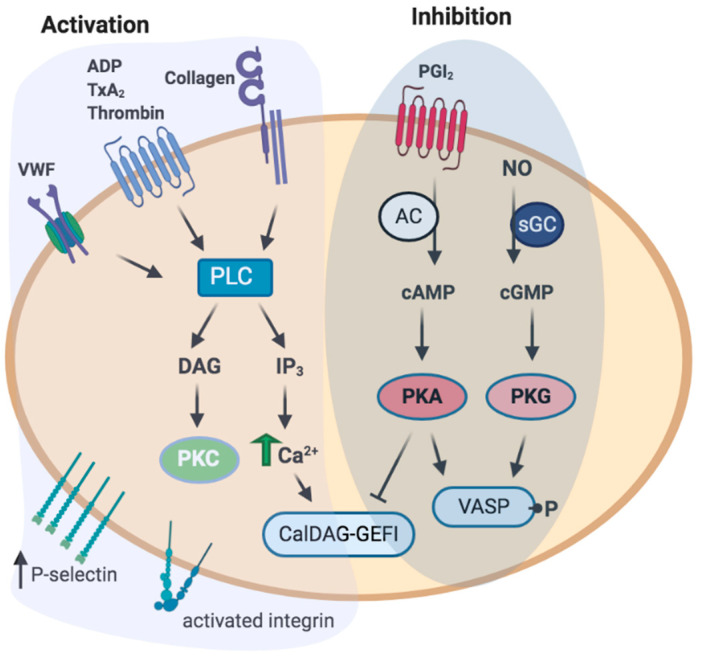
Platelet activation and inhibition. Stimulation with major platelet agonists (ADP, TxA2, thrombin, collagen, VWF) activates phospholipase C (PLC), and increases downstream signals, resulting in increased P-selectin expression and integrin activation. Major inhibitory signals include prostacyclin I2 (PGI2), and nitric oxide (NO), that increase cAMP, or cGMP, resulting in the activated protein kinase A (PKA), or protein kinase G (PKG), respectively. PKA and PKG phosphorylate multiple downstream targets, e.g., VASP, or CalDEG–GEFI and, thus, mediate negative effect on platelet activation. Tight regulation of signaling pathways leading to the activation or inhibition of platelets is required for their proper function. Abbreviations: VWF, Von Willebrand Factor; ADP, adenosine diphosphate; TxA2, thromboxane A2; PLC, phospholipase C; DAG, diacylglycerol; IP3, inositol trisphosphate; PKC, protein kinase C; PGI2, prostacyclin I2; NO, nitric oxide; AC, adenylyl cyclase; sGC, soluble guanylyl cyclase; PKA, protein kinase A; PKG, protein kinase G; VASP, vasodilator-stimulated phosphoprotein.

Receptor engagement triggers multiple intracellular signaling and cytoskeletal pathways that result in platelet activation (Figure 2). Collagen initiates platelet activation through GPVI, a member of the immunoglobulin family, which is coupled to Fc receptor γ (FcRγ). The cytoplasmic tail of FcRγ contains an immune receptor tyrosine-based activation motif (ITAM) that is phosphorylated by Src kinases [21]. On the other hand, soluble agonists (ADP, TxA2, thrombin) activate G-protein coupled receptors (GPCRs) that accelerate the platelet response. The activation of G-protein- and ITAM-coupled receptors will signal intracellularly via activation of phospholipase C (PLC, β or γ) that will generate second messengers inositol 1,4,5,-triphosphate (IP3) and diacylglycerol (DAG) from the membrane phospholipid phosphatidylinositol 4,5-bisphosphate (PIP2). IP3 will induce the release of Ca^2+^ from intracellular stores, while DAG will activate diverse protein kinase C isoforms [22]. The rise in Ca^2+^ propels several platelet responses, such as cytoskeletal changes, integrin activation, and degranulation. An increase in Ca^2+^ activates the small GTPase Rap1 via the guanine nucleotide exchange factor CalDAG–GEFI [23]. Subsequently, Rap1 recruits talin to the plasma membrane, thereby further activating integrin αIIbβ3. In turn, binding of activated αIIbβ3 to its ligands (e.g., fibrinogen) will transfer outside-in signals, regulating further cytoskeletal remodeling needed for full platelet spreading and clot retraction [24].

In certain pathological conditions, when the balance between platelet stimulatory and inhibitory (for details on inhibitory signaling see below) pathways is impaired, unbalanced clot formation (thrombosis) could lead to the occlusion of the vessels, and thereby lead to myocardial infarction or stroke (in arteries thrombi are rich in platelets) or to venous thromboembolism [25]. Conclusively, the tight regulation of platelet activation is crucial to ensure proper platelet function and prevent the formation of unwanted thrombi that could cause severe pathological outcomes.

### 1.4. Immunoregulatory Function of Platelets

**Figure 3:**
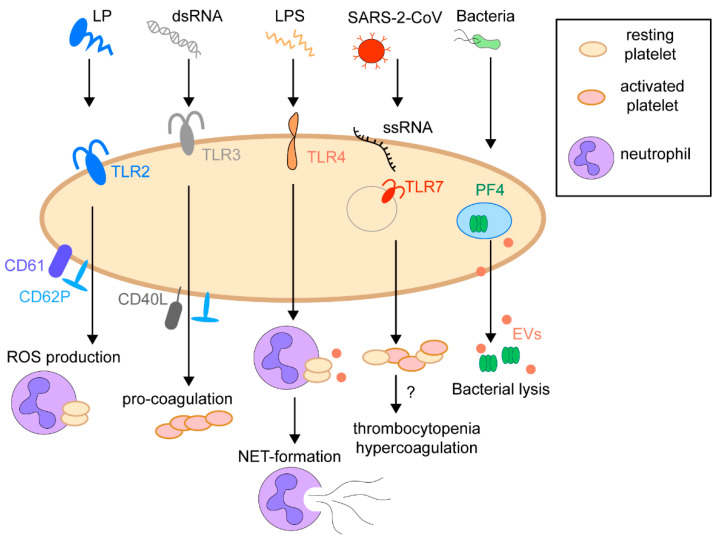
The immunoregulatory function of platelets. Platelets possess a set of pattern recognition receptors (PRRs) sensing pathogen-associated molecular patterns (PAMPs), such as lipoproteins (LP), lipopolysaccharides (LPS), nucleic acids, including double-strand RNA (dsRNA) and single-strand RNA (ssRNA).

In the last decade, there has been increasing evidence that platelets do much more than just maintain hemostasis and thrombosis. Many studies demonstrated their contribution to immunity and a pivotal role in developing inflammation, infections, and cancer [26,27,28,29,30,31]. Circulating platelets function as guardians sensing pathogens by a set of pattern recognition receptors (PRRs), including TLR, nod-like receptors (NLR), and C-type lectin receptors (CLR). These receptors sense pathogen-associated molecular patterns (PAMPs) such as lipoproteins, lipopolysaccharides (LPS), flagella, nucleic acids, proteins, or damage-associated molecular patterns (DAMPs) [32,33,34]. Ligation of platelets PRRs to the pathogens PAMPs effectively eliminates the pathogen or presents them to other cells of the immune system (Figure 3). For instance, stimulation of platelet TLR2 increased the P-selectin surface expression, activation of integrin αIIbβ3, generation of reactive oxygen species, and formation of platelet–neutrophil heterotypic aggregates in human blood [35]. The activation of platelets TLR4 receptor by bacterial LPS is still controversial. On the one hand, it activates MyD88/cGMP-dependent protein kinase signaling, which initiates the platelet secretion and potentiates platelet aggregation [36]. On the other hand, platelets activated by LPS have been described to form aggregates with polymorphonuclear (PMN) cells and inhibit the weak platelet activation by ADP [37]. The activation by LPS also promotes platelet–neutrophil adhesion and rapid formation of neutrophil extracellular traps (NETs) to eliminate bacteria from the bloodstream [36,38,39]. Similar to TLR2, platelets primed with endosomal receptors TLR3 agonists expose P-selectin/CD40L on the surface and have enhanced procoagulant responses to thrombin or other traditional agonists [40]. TLR7, located in the platelets endolysosomes, senses various RNA viruses such as influenza and severe acute respiratory syndrome coronavirus 2 (SARS-CoV-2) [41,42,43]. The activation of platelets through TLR7 can decrease platelet count (thrombocytopenia) and cause hypercoagulation. The detailed mechanism of this activation is not yet investigated [44,45]. Although numerous studies indicated that platelets express the full set of PRRs, there is a limited evidence of their presence in MS-based proteomics data [46,47]. This is probably due to the methodological limitations, membrane nature, and the multiple post-translational modifications of these receptors, limiting its detection by mass spectrometry. It could also be because some TLRs are expressed mainly after platelet activation, while first comprehensive platelet proteome analyses with deep coverage were performed on resting platelets [32,46,47].

In different disease settings, platelets have been identified as both friends and foes. On the one hand, they could be a vehicle for disseminating viral infections, such as influenza, hepatitis C, dengue virus, or HIV [30,44,48,49,50]. On the other hand, they can kill pathogens directly by wrapping, activated trapping, and producing anti-microbial peptides or indirectly through activation of other immune cells. One of the most abundant platelet proteins with estimated copy numbers ≈ 600,000/cell is platelet factor 4 (PF4), released from α-granules, binds the membrane of Gram-negative and Gram-positive bacteria, leading to their elimination [47,51,52]. The interaction of platelets with pathogens causes rapid platelet activation. Following activation, platelets secrete pro- and anti-inflammatory molecules and several types of platelet-derived extracellular vesicles, recruiting neutrophils, macrophages, and dendritic cells to the site of infection [28]. For example, infection with dengue virus activates platelets via CLEC2, a tyrosine kinase (Syk)-coupled C-type lectin receptor, to release extracellular vesicles, which activate CLEC5A and TLR2 on neutrophils and macrophages, thereby inducing NET-formation and proinflammatory cytokine release [53]. Platelets are also activated by interaction with the spike protein of SARS-CoV-2. This interaction directly stimulates platelets to release coagulation factors, secrete inflammatory factors, and form leukocyte–platelet aggregates. Polyphosphates (inorganic polymers composed of phosphate units) are exposed on procoagulant platelets and trigger coagulation by activating the intrinsic pathway of coagulation. Recently, the phosphate exporter XPR1 was identified as the first regulator of platelet polyphosphate with critical implications for thrombosis [54,55]. Platelets of COVID-19 patients showed increased activation and aggregation, partly attributed to the increased MAPK pathway stimulation and TxA2 generation [56,57]. There appears to be increased rates of thrombotic events including microvascular thrombosis, venous thromboembolic disease, and stroke in COVID-19 patients [58].

Accumulating evidence suggests that platelets interact with neutrophils and monocytes and function as antigen-presenting cells (APCs) to prime T-cell lymphocytes [27,59,60,61,62]. The first drafts of human and mouse platelets proteome clearly showed that platelets are fully equipped for antigen presentation. They possess proteasome proteins, a transporter of antigen peptide (TAP), and major histocompatibility complex (MHC) class I molecules themselves [46,47]. Lately, it was shown that platelets acquired all machinery for antigen-presenting through megakaryocytes [63]. Even though megakaryocytes are found to express both MHC class I and II molecules, the platelets proteome and experimental evidence show only active MHC class I presentation [59,63,64]. Interestingly, the declined level of MHC class I molecules on the surface could indicate platelet aging [65].

## 2. Platelets Proteomics

**Figure 4:**
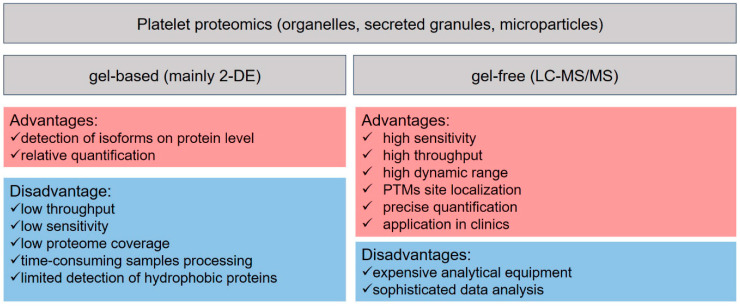
Advantages and limitations of basic methods in proteomics.

Two decades ago, the term platelet proteomics was mainly referred to two-dimensional gel electrophoresis (2-DE), where the proteins are separated according to their isoelectric point in the first and by molecular weight in the second dimension [66,67,68]. This technology was used to identify proteins and mapping protein phosphorylation of rested and activated platelets, the composition of platelets subcellular organelles, e.g., lipid rafts, membrane, secreted granules, and platelet microparticles [69,70,71,72,73]. Additional comparison of proteins staining or pre-labeling the proteins from different biological samples and mixing them before separation allowed for rough relative quantification and monitoring the platelet differences at diverse physiological and pathophysiological conditions [69,70,74,75,76,77]. Despite popularity gained in the platelet research community and multiple studies for analysis of platelets and their suborganelles, 2-DE has some limitations, such as detecting and identifying low-abundant and highly hydrophobic proteins (Figure 4). The 2-DE platelet studies are comprehensively reviewed elsewhere [78,79,80]; therefore, in the following chapters, we will focus mainly on the gel-free liquid chromatography (LC) mass spectrometry (MS)-based proteomic works and frontier technologies in MS for future perspectives in studying platelet biology.

### 2.1. Basics of LC-MS-Based Proteomics

**Figure 5:**
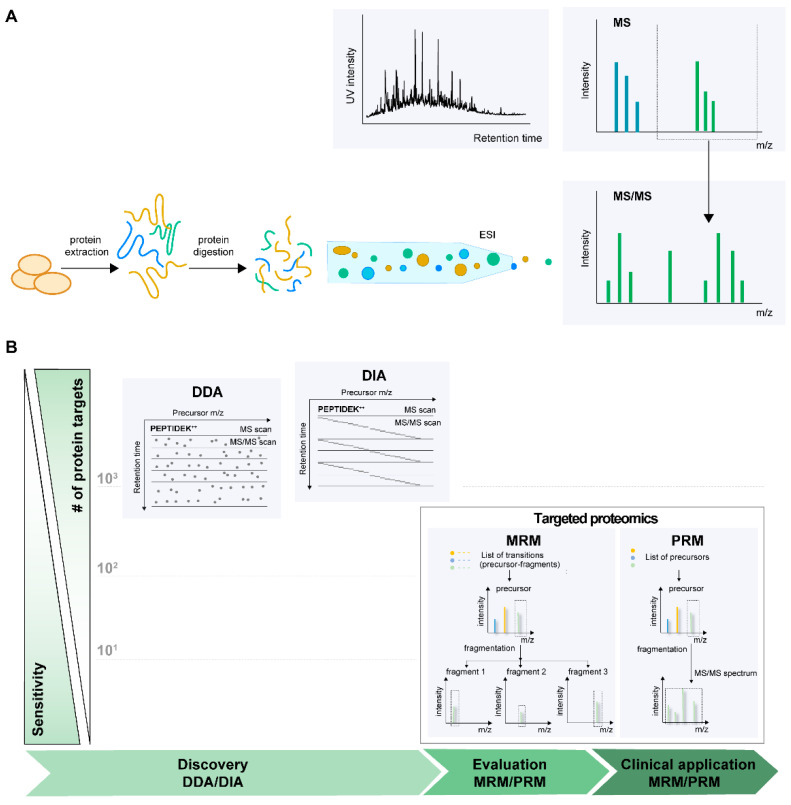
(**A**) LC–MS/MS based bottom-up proteomic workflow. Platelets isolated from blood are subjected to lysis, enzymatic digestion, separation of peptides on a reversed-phase LC column, ionized (ESI), and mass analyzed (MS). (**B**) Modes of MS data acquisition. Three main strategies for data acquisition: data-dependent acquisition (DDA), data-independent acquisition (DIA), and targeted acquisition by multiple or parallel reaction monitoring (MRM or PRM). The DDA and DIA are aimed to analyze global proteomic changes, whereas the targeted methods focused on monitoring preselected target proteins with high precision and accuracy.

Current MS-based proteomics approaches rely on two main ionization techniques e.g., matrix-assisted laser desorption/ionization (MALDI) [81] and electrospray ionization (ESI) [82]. Both methods are frequently used in proteomics research, but ESI–MS has garnered popularity in the recent past. Furthermore, proteomics workflows can be broadly classified as top-down and bottom-up to analyze intact proteins and peptides, respectively. In a typical bottom-up proteomics setup, the biological sample, e.g., platelets, are lysed and subjected to enzymatic (mostly trypsin) proteolysis to generate peptides. To minimize this mixture’s complexity, a high-performance liquid chromatography (HPLC) is used upfront in combination with ESI as an interface and coupled to the MS. The peptide ions enter the MS for subsequent mass analysis and detection. MS analysis of peptides can be further divided into peptide mass fingerprinting and tandem MS (MS/MS); the latter approach will be explained in detail (Figure 5A). Presently, there are three main modes for tandem MS data acquisition: data-dependent acquisition (DDA), data-independent acquisition (DIA), and targeted acquisition by selected or multiple reaction monitoring (SRM/MRM), and recently developed parallel reaction monitoring (PRM). The DDA and DIA are mainly employed in the so-called discovery phase, aiming to analyze global proteome profiles or changes at different time points or conditions in a relative manner. Targeted methods, also called hypothesis-driven methods, are directed to monitor from one to a couple of hundreds of proteins of interest with high selectivity, specificity, and sensitivity [83]. Typically, the shift from discovery to targeted phase leads to a reduction in the number of protein candidates, i.e., from several thousand to a few dozen, which corresponds to the detection and validation of biomarkers in the clinical samples (Figure 5B).

In DDA, the precursor ions, usually the top 10–20 peptides per duty cycle of MS instrument (~1 s), are consecutively selected from a full mass MS1 scan for fragmentation, and the information on product ions is acquired during MS2 scans. Precursor ions (MS1) are fragmented in a semi stochastic manner in order of decreasing intensity, and database search is then performed on the MS2 fragmentation spectra to identify the sequence of corresponding MS1 precursor ion [84]. The technology was applied to detect and quantify proteins and their post-translational modifications, creating a comprehensive map of platelet cytosolic and membrane proteins [46,47,80,85]. For quantifying differences in proteome among biological samples or during the time course of platelet activation, isobaric labeling, such as tandem mass tags (TMT) or isobaric tags for relative and absolute quantitation (iTRAQ), are applied [86,87,88]. Both types of tags use stable isotope-labeled molecules that can be covalently bound to the N-terminus and side-chain amines of proteins or peptides. Each label has the same molecular weights and chemical properties and comprises a reporter and balanced groups. The peptides of the same sequence, but labeled with different isobaric tags, could be differentiated during fragmentation by yielding reporter ions with different masses [89]. The labeling allows for multiplexing of samples on protein or peptide levels, reduces experimental variation, and, thus, quantification errors. The prefractionation of multiplexed samples additionally increases the total coverage of platelets proteome. The methodology was applied to study ADP-mediated signaling [90,91] and cAMP/PKA-dependent signaling in platelets [92]. It was a main method of choice to study platelets of patients with Scott syndrome [93], gray platelet syndrome [94], Albright hereditary osteodystrophy syndromes [95], and type I Glanzmann thrombasthenia [96]. Alternatively to TMT and iTRAQ, unlabeled samples can be compared by monitoring the relative abundance of peptides assessed by comparing either intensity or the integrated peptide peak area in extracted ion chromatogram (XIC) using special software for data processing. Despite the robustness of the method, high sequencing speed of new generation of MS instruments, and well-established pipelines for data analyses, DDA’s stochastic precursor selection leads to inconsistent detection of peptides, especially those from low-abundant proteins. This data acquisition mode results in missing peptide identification and reduces the number of quantifiable proteins [97]. Despite the lower reproducibility at the low abundance protein level, the DDA is still the main method of choice for most platelet studies.

The DIA is a newly emerging alternative to DDA strategy, which has higher reproducibility of identification, improved sensitivity, and quantification accuracy, shown on peptides and phosphopeptide levels [98,99]. In DIA, all ionized precursors within a specified mass range are fragmented in consecutive survey scans. Fragment ion spectra for all the precursors in a predetermined isolation window are acquired [100,101]. Instead of databases of sequenced organisms for DDA, the DIA typically requires information about peptides to detect proteins, named peptide query parameters (PQPs). PQPs include chromatographic elution time, called retention time (RT) of selected peptides and their MS characteristics, such as charge state and m/z information of precursor ions and the four to six most intensive fragmented ions.

The third common strategy-targeted analysis is applied when the proteins of interest are predetermined and need to be quantified across multiple samples with a high degree of reproducibility and quantitative accuracy [102]. Like in DIA, the pre-existing information about proteotypic peptides, including RT, charge, and m/z of precursor and fragmented ions, is required. Typically, this information is acquired from spectral libraries, the informational source about proteotypic peptides of interest. There are two main types of targeted assays depending on instrumentation: MRM performs only one precursor ion/product ion transition in time, while PRM analyzes all product ions derived from a precursor ion with high resolution and mass accuracy. To date, PRM was mainly used for validation of DDA discovery studies on platelets, such as validation of regulated proteins from Scott patients platelets [93]; Glanzmann thrombasthenia patients [96]; alteration of specific phosphosite phosphorylation in ADP activated platelets [90]; monitoring the coactivation of multiple small GTPase isoforms in response to agonists [103]; reduction of apoptosis-related proteins in the bone marrow of immune thrombocytopenia patients [104]. The study applying PRM combined with isotope-labeled peptide standard quantified 99 proteins related to platelet activation and functional disorders [105]. The PRM combined with the SILAC-labeled protein standard was also established for quantification of frataxin, a platelet protein marker for autosomal recessive disease—“Friedreich’s ataxia” [106].

The recent introduction of a new type of mass spectrometer with ion mobility as an additional dimension for peptide separation brings much promise in proteomic research in general and particularly in platelet signaling biology. Introduced by Bruker, trapped ion mobility spectrometry (TIMS) coupled to time-of-flight (TOF) mass analyzer in combination with a new scan mode for data acquisition called Parallel Accumulation–Serial Fragmentation (PASEF) allows increasing the sequencing speed by approximately tenfold without loss in sensitivity [107,108]. Following electrospray ionization, ion mobility discriminates gas-phase peptide ions by their size and charge before mass analysis. PASEF does not only increase the number of peptide identification per LC–MS run to over 6000 HeLa proteins/120-min LC run, which, applicable to platelet proteome, theoretically could allow achieving (almost) full coverage from a low amount of samples, but also allows for accurate separation of isomers, e.g., phosphopeptides, and should facilitate the localization of phosphosites, crucial for studies on activation and inhibition in platelet signaling.

### 2.2. Sample Preparation as a Crucial Step for Accurate Platelets Proteome Research

The sample preparation for accurate proteomic analysis of platelets is still a challenging task. The purity, number, and activation status of platelets are crucial for reliable proteome analysis. The first comprehensive human platelet proteome, comprising almost 4000 unique proteins, was performed from 10^9^ platelets yielding 1.5 mg of proteins [47]. Current advances in MS greatly improve the sensitivity and reduce the amount of required starting material until almost one cell or a few ng of proteins [109,110,111]. Considering that the physiological normal platelet count is between 150,000 and 350,000 platelets per microliter [112], only a few microliters of blood should be sufficient for MS-based/platelets-based analysis in the future. The separation of platelets from whole blood has improved during the last decades. Most of the standard procedures for platelet preparation are based on low-speed centrifugation, where the whole blood is separated by different densities of various blood components; herein, the small and light platelets remain suspended in the liquid plasma [113,114]. Due to the platelet’s sponge-like open canalicular membrane system, the contamination with plasma components cannot be completely excluded. In 2014, in an initial proteome study on mouse platelets, M. Zeiler et al. monitored protein abundance profiles across different purification steps that distinguished true platelet proteins from plasma, leukocytes, and erythrocyte contaminants [46]. The clustering of proteins with different stages of purification processes allowed for detecting more than 200 contaminants. The majority of them belong to highly abundant plasma proteins (apolipoproteins, complement factors) or components of erythrocytes [46]. The OptiPrep^TM^ density gradient centrifugation technique was reported to have 99.99% platelet purity and low leukocyte contamination, recovering more than half of the platelet population, but has not been applied for proteomic studies [115]. Alternatively to centrifugation techniques, microfluidic platelet preparation could be applied. A fully automated procedure for analyses of platelet transcriptome, published in 2020, provides high yield and purity (>99%) with lower platelet activation [116]. With protein low-binding tubing material, the method could also be applicable for proteomic sample preparation. The additional controls, ensuring complete platelet lysis and digestion [117,118], minimal loss of peptide during sample preparation, and automation for the analysis of hundreds or thousands of samples should be established for high-throughput studies [119]. Finally, quality control systems of mass spectrometry-based proteomics data acquisition should be introduced in the labs dealing with platelets’ biomedical and translational applications, e.g., a cloud-based quality control system [120] or web-based applications [121,122].

### 2.3. Strategies to Study Protein Post-Translational Modifications in Platelet Biology

Post-translational modifications (PTMs) refer to the modification of proteins after biosynthesis and are known to control multiple biological functions, including protein activity, folding, localization, and interaction with other biomolecules. PTM is described as an attachment, removal, exchange, or rearrangement of functional groups to amino acid side chains and protein N- and C-termini [123,124,125]. Although more than 400 PTMs are described, their physiological roles are only partially unraveled [123,126,127]. For platelets, the intensively studied PTMs are phosphorylation, ubiquitylation, and proteolysis. Less attention is paid to glycosylation, acetylation, and palmitoylation. Of special interest is also the interplay of platelet PTMs during activation.

#### 2.3.1. Phosphorylation

Phosphorylation is one of the most occurring and well-studied platelet PTM. Modulation of signaling pathways during platelet activation significantly changes the phosphorylated proteins’ landscape that directs platelet function. The processes of protein phosphorylation and dephosphorylation are catalyzed by kinases and phosphatases, respectively. The phosphorylation can occur on serine, threonine, and tyrosine residues through phosphoester bond formation. Rarely histidine, lysine, and arginine could be modified through phosphoramidate bonds side. The phosphopeptides could be detected directly in peptide digest only with very low extend [87]; therefore, multiple phosphopeptide enrichment strategies were employed to remove the bulk of unphosphorylated peptides and, thus, increase the sensitivity of detection. The most common methods employed for phosphopeptides enrichment include Fe^3+^- immobilized metal ion affinity chromatography (IMAC) [73], metal oxide affinity chromatography using titanium dioxide (TiO_2_) or zirconium dioxide (ZrO_2_) [128,129]; electrostatic repulsion–hydrophilic interaction chromatography (ERLIC) [130], strong cation and anion exchange chromatography (SCX, SAX), and enrichment of phosphorylated proteins by immunoprecipitation [131].

A peptide with the same sequence could undergo different combinations of phosphorylations. The accurate localization of these sites was a challenge in phosphoproteomics for many years. Targeted proteomics overcame this complication and allowed for accurate localization of phosphorylation sites and calculation of phosphorylation ratios [132].

Enormous progress in the field was establishing phosphoproteome DIA studies (PhosphoDIA), allowing for routine quantification of ~7000 phosphopeptides of Hela digest in just 15 min of LC–MS/MS analysis. DIA-based phosphoproteomics achieves an order of magnitude broader dynamic range, higher reproducibility of identification, and improved sensitivity and accuracy of quantification compared to state-of-the-art DDA-based phosphoproteomics. The phosphosite localization algorithm based on peptide-centric analysis utilizing information is established for cell lines, but still not applied for platelet studies [98].

##### Identification of Key Players of Platelet Activation Pathways by Proteomics Approaches

The first phosphoproteomic studies based on the phosphopeptides enrichment method revealed new phosphorylation sites in resting [73,133] and thrombin-stimulated platelets [133]. Further proteome analysis was done upon platelet stimulation of GPVI receptor with collagen-related-peptide (CRP) [134], or by activating with a monoclonal antibody specific for GPVI [135]. Later, changes in 214 unique phosphotyrosine sites and oligophenin-1 (OPHN1) were determined as one of the key regulators of platelet filopodia formation upon GPVI stimulation [136].

Platelet signaling cascades were studied with TRAP (thrombin-receptor activated peptide, activates PAR1 receptor) stimulation of human platelets by nanoLC-MS/MS that revealed for the first time phosphorylation of the regulator of G protein signaling (RGS) proteins, GTPase-activating proteins that regulate G-protein-coupled receptors [137].

ADP receptors are important drug targets, especially in preventing thrombosis in high-risk patients [138]. Temporal quantitative phosphoproteomics of ADP-stimulated platelets showed that phosphorylations occur within 10 s and are transient in most cases. The study suggested several central hubs (e.g., CalDAG–GEFI, phosphodiesterase type III) that represent control points of platelet activation and inhibition since they were inversely phosphorylated by ADP and cAMP-elevating compound iloprost (a stable prostacyclin analog) [90]. A recent study utilized fluorescence 2-D Fluorescence Difference Gel Electrophoresis and analyzed the human non-secretory platelet proteome after TRAP and ADP activation, cAMP/protein kinase A-mediated inhibition (prostaglandin I2 or CTAD formulation), and compared it to resting platelets [139]. The study discovered that upon platelet activation or inhibition, proteome changes are mainly related to the phosphorylations (induced by both activation and inhibition on different phosphosites) and that inhibition induces qualitatively and quantitatively stronger changes (explained in more details below) than platelet activation. In addition, new and more robust potential biomarkers were suggested to more effectively discern platelet activation, such as phosphorylated integrin-linked protein kinase (ILK) and pleckstrin (PLEK) instead of P-selectin [139].

##### Identification of Key Players of Platelet Inhibitory Pathways by Proteomic Approaches

Cyclic nucleotides (cAMP and cGMP) and corresponding protein kinases, protein kinase A (PKA), and protein kinase G (PKG) are the main mediators of platelet inhibitory pathways [140,141]. Endothelial cells release short-lived mediators such as a nitric oxide (NO) and prostacyclin (PGI2), which activate soluble guanylate cyclase (sGC) and adenylate cyclase (AC), respectively (Figure 2). First quantitative platelet proteomic analysis revealed expression of three isoforms of AC (ADCY3, ADCY5, and ADCY6), four PKA regulatory (PKARIA, PKARIB, PKARIIA, and PKARIIB), and three catalytic subunits (PKACA, PKACB, and PKACG) [47]. Concerning NO/sGC/PKG pathway, none of the known NO synthase isoforms were found to be expressed in platelets [142,143], and sGC (sGCα1, and sGCβ1) is the only enzyme responsible for cGMP synthesis in platelets [144]. From three known mammalian PKG isoforms, only PKG1β is expressed in platelets [47]. The development of phosphoproteomic techniques allowed the identification of new PKA/PKG substrates directly involved in different platelet inhibitory pathways. By analyzing phosphorylated proteins of resting human platelets, 564 phosphorylation sites from more than 270 proteins were identified, and among them, 23 proteins contain putative PKA/PKG phosphorylation consensus (R/K|R/K|X|S/T) sites [73]. However, these data needed additional validations, and even in the comprehensive review on PKA/PKG signaling in platelets, only 15 proteins are mentioned as established substrates of these kinases [141].

Future progress on establishing new platelet PKA/PKG substrates was achieved by analysis of platelets upon stimulation of the cAMP/PKA pathway using a quantitative phosphoproteomic approach [90,92]. Almost 300 proteins with changed phosphorylation patterns, among which only 137 had PKA phosphorylation consensus sequence, were identified by analysis of iloprost-stimulated platelets. The most important message of these data is that more than half of differentially phosphorylated proteins are not direct PKA substrates, indicating that PKA-induced platelet inhibition is a complicated multistep process comprising not only direct PKA substrates, but involving also many other protein kinases and phosphatases. Most of the new PKA substrates identified by phosphoproteomic are involved in different intracellular platelet inhibitory pathways including inhibition of calcium signaling, regulation of Ras/Rho family small GTPases, and reorganization of the actin cytoskeleton. Several proteins involved in platelet calcium signaling, including the IP3 receptor, IRAG, and TRP6, were already known as PKA/PKG substrates and their phosphorylation is directly connected with inhibition of calcium mobilization [145,146]. Phosphorylation patterns of several other proteins potentially involved in calcium signaling were found regulated by PKA. Interestingly, phosphorylation of calcium release-activated calcium channel protein 1 (Orai1) at Thr295 was decreased by PKA activation. However, whether this was connected to the regulation of calcium release from intracellular stores is not known [92]. Another finding of this study is related to the phosphorylation of bridging integrator 2 (BIN2) protein at Ser259. After recently showing that BIN2 is an interaction partner of STIM1 and IP3R it was further investigated that platelets deficient in BIN2 had reduced calcium release and influx upon agonist stimulation [147]. Whether phosphorylation of BIN2 by PKA is directly connected with inhibition of calcium signaling in platelets warrant future analysis.

Small GTPases function as a molecular switch by cycling between active GTP-bound and inactive GDP-bound states, which is controlled by GTPase activating proteins (GAPs) and guanine nucleotide exchange factors (GEFs). RhoA, Rac1, Cdc42, and Rap small GTPases are activated in agonists-stimulated platelets and inhibited by PKA/PKG. Previously phosphorylation of RhoA at Ser188 by PKA/PKG was proposed as one of the mechanisms of its inhibition [148,149,150]. However, in the phosphoproteomic analysis of PKA/PKG-signaling direct phosphorylation of RhoA at Ser188 in platelets was not confirmed [90,92] and this phosphorylation is probably connected with unspecific binding of phospho-specific antibodies to some other protein with similar molecular weight [151]. Future detailed analysis of RhoA inhibition by PKA/PKG will provide evidence that it directly correlates with the phosphorylation of the RhoA-specific GTPase-activating protein Myo9b at Ser1354 and the guanine nucleotide exchange factor GEF-H1 at Ser886. Phosphorylation of Myo9b enhances its GTPase activity thereby reducing active RhoA (RhoA–GTP) levels and GEF-H1 phosphorylation inhibiting GEF function [151].

Moreover, inhibition of Rac1 activity by PKA/PKG in platelets is connected with the phosphorylation of Rac1 specific GAPs and GEFs. PKA and PKG phosphorylate ARHGEF6 at Ser684 and ARHGAP17 at Ser702, which results in a reorganization of signaling complexes involving CIP4 and 14-3-3 proteins and inhibition of Rac1 activity [152].

Rap1 itself, as well as Rap1GAP2, and CalDAG–GEF1, which controls Rap1 activity, are established PKA/PKG substrates [153,154,155]. Rap1B is phosphorylated by PKA at Ser179 [153] and Rap1GAP2 is phosphorylated at Ser7 [156]. However, the kinetics of Rap1 phosphorylation and functional analysis of Rap1GAP2 phosphorylation show that these events are not directly related to PKA-mediated Rap1 inhibition. CalDAG–GEF1 contains four putative phosphorylation sites (Ser116/117, Ser147, and Ser587), and after PKA activation of platelets, strong phosphorylation (more than 35 times increase of phosphate incorporation) was detected on Ser587 by a quantitative phosphoproteomic analysis, which correlates with inhibition of integrin αIIbβ3 activation and platelet aggregation [155]. Thus, the analysis of platelet proteins phosphorylated by PKA [90,92] already helps to identify new important players in the platelet inhibitory pathway, and the herein identified proteins with altered phosphorylation status will contribute to characterize platelet inhibition in more detail.

Analysis of sGC/cGMP/PKG-induced phosphorylated proteins was the next important step in characterizing platelet inhibitory mechanisms. After incubation of platelets with different NO donors and a direct sGC stimulator riociguat they were analyzed by quantitative phosphoproteomic methods ([140] and our still ongoing project) to unravel platelet inhibitory mechanisms. Increased phosphorylation upon PKG stimulation was detected on more than 150 proteins, including known PKG substrates like VASP, PDE5, Rap1GAP2, and others, and, similar to PKA effects, decreased the phosphorylation of more than 60 proteins. PKA and PKG have an overlapping substrate specificity and all the above-mentioned functionally validated PKA substrates are targets of PKG.

It is important to mention that although PKA/PKG is the most powerful platelet inhibitory pathway, there is increasing evidence for other platelet-derived inhibitory mechanisms. Several intracellular receptors including glucocorticoid receptors (GRs), peroxisome proliferator-activated receptors (PPARs), liver X receptor (LXR), retinoid X receptor (RXRs), and immunoreceptor tyrosine-based inhibitory motif (ITIMs) containing receptors such as PECAM-1, CEACAM1/2, and G6b-B are also important modulators of platelet inhibitory pathways [157,158,159]. Application of novel proteomic approaches will shed new light on understanding the highly complex platelet activatory and inhibitory machinery and help in developing new strategies for antiplatelet drugs.

#### 2.3.2. Ubiquitylation and Proteolysis in Platelet Biology

An additional layer in platelet regulation is the ubiquitin-proteasome system, which maintains cellular protein homeostasis, regulates signal transduction cascades, and supplies MHC class I molecules with peptides for antigen presentation [64]. Platelets express standard and immunoproteasome subunits and display three protease activities, caspase-like, trypsin-like, and chymotrypsin-like activity, executed by the catalytically active β subunits [64,160]. To undergo proteasome degradation, proteins first need to be marked by ubiquitination, a PTM where a small, highly conserved protein called ubiquitin (Ub) is covalently bonded to a lysine residue. Ubiquitin itself encloses seven lysine residues, where other ubiquitin moieties can bind, resulting in branched poly-Ub, or multi-Ub chains [161]. There are several techniques for proteomic identification of ubiquitination sites. The most common are immune affinity enrichment of Ub- proteins and di-glycine remnant detection. The first method utilized the Ub-specific specific antibody. The second method is based on the idea that after digestion of ubiquitinated proteins with trypsin, on the place of previously ubiquitinated lysine remains a short di-glycine remnant. The di-glycine remnant leads to a peptide mass shift of (monoisotopic, 114.0429 Da) and can be used to localize the ubiquitin attachment site [162]. Anti-K-ε-GG (di-glycine remnant) antibodies can additionally enrich peptides with a diglycine remnant motif before applying LC–MS/MS identification [163]. Several functional studies elucidated the role of ubiquitination in platelet signaling. For example, spleen tyrosine kinase (Syk) is rapidly ubiquitinated upon activation of platelets by collagen, CRP, and convulxin, which lead to the increased activity of this kinase [164]. A more detailed investigation of platelets stimulated with CRP was performed by utilizing di-glycine remnant technology, confirming multiple ubiquitinylation of Syk, and additionally ubiquitination of the components of GPVI signaling pathway [165]. So far, it is the first MS proteomic study for platelet ubiquitination.

Understanding the mechanisms of proteolytic events in platelets and coagulation cascade activated by platelets is also crucial, since protein proteolysis could lead to activation, deactivation, or complete alteration of protein function. The proteolysis can be studied by analyzing N- or C- termini and, similar to other PTM, requires an enrichment step for more comprehensive coverage [166]. N-terminus selection, combined with charge-based fractional diagonal chromatography (ChaFRADIC) was mainly employed for platelets [167]. The application of the ChaFRADIC demonstrated that activated platelets from Scott-syndrome patients have enrichment of caspase-mediated proteolytic cleavages [93]. In the context of cellular signaling, the simultaneous analyses of phosphorylation, ubiquitination, and proteolysis events during platelet activation would be of special interest.

## 3. Platelets Proteomics in Health and Disease

The early studies on platelet proteomics comprehensively describe the composition and copy numbers of mouse and human platelets, analyzed phosphorylation, and other PTM in a steady-state, and stimulated with different agonists [46,47,85]. The proteomic content of granules released by platelets during activation, collectively named “platelet releasate”, was also defined and quantified [8]. A comprehensive map of human platelets and inter- and intra-donor variation analysis showed the stability of ≈ 85% of the platelet proteome [47,74]. These provided the basics to study alteration of platelets caused by different diseases and, ideally, for potential pharmaceutical targeting of the underlying signaling pathways.

Platelets represent a critical factor in developing cardiovascular diseases, the leading cause of death in the industrialized world. Arteriosclerosis of the coronary arteries is the underlying disease for acute and chronic coronary syndromes and platelets contribute significantly to the progression of both entities. The underlying signal transduction remains, however, incompletely understood and tools for the diagnosis and prognostication of disease progression are urgently needed. Scarce platelet proteomic evidence is currently available. When comparing patients with acute and chronic coronary syndromes, proteomic studies revealed that six out of 400 proteins studied were differently expressed [168]. Additional studies assessed protein expression and small cohorts of 10-30 patients presenting with acute vs chronic coronary syndromes. It was demonstrated that the differentially regulated proteins contribute to cell assembly processes, organization and morphology, which are essential for platelet activation [169]. While these previous studies indicate that platelet activation differs between specific cardiovascular diseases, large scale validation studies are required to assess whether platelet proteomics may be a useful tool in cardiology care and clinical routine. With latest technology, the unbalanced platelet reactivity and potential subsequent thrombus formation could be assayed by monitoring the protein abundance in “platelet releasates”. Comparative proteomics of “platelet releasates” has allowed differentiating acute coronary syndrome versus stable coronary artery disease [170]. A proof-of-concept study applied on a small cohort of patients with lung or pancreas cancer showed that platelet proteome harbors differentially expressed proteins associated with early-stage cancer and identifies platelet proteins as a new source of potential biomarkers [171].

Much attention was put to rare genetic diseases associated with a bleeding disorder. The mechanisms of phenotypical changes in platelets of patients with Scott syndrome were investigated by a comprehensive proteome, phosphoproteome, and N-terminome analysis between resting and stimulated Scott and control platelets. Multipronged proteomic profiling of Scott platelets discovered the reduction of calpain-induced cleavages of cytoskeleton-linked and signaling proteins, and provided detailed insight into their protection against detrimental Ca^2+^-dependent changes that are normally associated with phosphatidylserine exposure [93]. Platelets of patients with Gray platelet syndrome compared to healthy donors revealed a diminished abundance of “alpha-granules” and “releasate” proteins and increased amounts of proteins normally resident in neutrophil granules [94]. Abnormality of platelets in Wiskott-Aldrich syndrome (WAS) was studied in a mouse model lacking WAS protein. The proteome profile of platelets showed inhibition of several metabolic pathways and enhancement of ubiquitination and proteasomal activity that increases antigen processing, contributing to triggering of autoimmunity. Studies performed in mice were, in part, confirmed in WAS patients [172].

Besides many already published studies describing a key role of platelets in cardiovascular disease by utilizing mass spectrometry based OMICS technologies an upcoming are of interest is platelet dysfunction during kidney disease.

Chronic kidney disease (CKD) is associated with disturbances in platelet function, leading to both thrombotic complications and bleeding [173,174]. Platelet dysfunction is thought to be the major cause for these events and contributing to the high morbidity and mortalities rates in CKD. Potential contributing factors involve platelet glycoproteins GPIIb/IIIa, ADP and serotonin release as well as metabolic disturbances of arachidonic acid and prostaglandins [173]. Recently, the gut microbiota as a source of uremic toxins has shown to be a risk factor for thromboembolic complications [175]. These include metabolites of dietary tryptophan (indoxyl sulfate (IS), indole-3-acetic acid and kynurenine (KYN)), phenylalanine/tyrosine (p-cresol sulfate (PCS), p-cresol glucuronide (PCG), phenylacetylglutamine (PAGln)), and choline/phosphatidylcholine (trimethylamine N-oxide (TMAO)) [176]. Uremic toxins have been shown to effect endothelial cells, vascular smooth muscle cells, macrophages and platelets, leading to increased inflammation, platelet activation and aggregation [177], e.g., via the release of endothelial microparticles [178], production of reactive oxygen species (ROS) [178], or decreased production of nitric oxide [179]. Unraveling contributing factors of platelet dysfunction by mass spectrometry-based proteomics for diagnostic and prognostic purposes would greatly contribute to identification of risk factors and treatment options due to its multifactorial composition and disease stage-associated and interpatient variability.

## 4. Platelets Proteomics in Transfusion Medicine

Platelet transfusion is a lifesaving medical procedure, normally used to restore platelet counts in thrombocytopenic patients and patients with platelet dysfunctionality. Platelets for transfusions are normally prepared either by centrifugation of the whole blood or by a method called plateletpheresis-a type of apheresis, where the platelets are separated from blood obtained from a single donor while returning to a donor the red blood cells (RBCs), white blood cells (WBCs), and plasma components. Platelet collection, processing, and storage could affect platelets’ structure and function, causing multiple platelet storage lesions (PSLs) and bacterial contamination [180,181]. During prolonged storage, platelets and storage medium undergo drastic changes, e.g., the platelets decreased response to agonists, changed the metabolism, and increased production of reactive oxygen species, leading to platelet activation and dysfunction [182,183,184]. In recent years, the mechanism underlying PSLs started to be investigated more intensively by the proteomic approach. The early study utilized differential in-gel electrophoresis (DIGE) combined with mass spectrometry showed the alteration of septin and gelsolin during platelet storage [185]. With the advancement of technology, the proteomic studies employing label-free quantification revealed that prolonged storage (13–16 days) downregulates proteins involved in platelet degranulation, secretion, exocytosis, and, at the same time, upregulates the α-2-macroglobulin, glycogenin, and Ig μ chain C region [186].

Likewise, proteomics is actively employed to characterize temperature-induced platelet alterations. Not widely known, platelets, unlike other blood cells, rapidly leave the circulation if refrigerated before transfusion [154]. The clearance occurs through triggered surface up-regulation of neuraminidases, which perform the vWF-receptor complex’s desialylation, specifically, the GPIbα subunit leading to GPIbα-clustering and rapid platelet clearance by liver phagocytes [154,187,188]. Although cryopreservation slows down the metabolism, reduces the risk of bacterial contamination, and is therefore proposed as a prominent alternative to the current storage standard at 22 °C, its application is still under debate since the freeze-thawing-induced activation of platelets may promote the risk of thromboembolic events [189]. Wang et al. compared the proteomic signature of platelets stored at 22 °C, 10 °C, and −80 °C and concluded that different conditions caused different PSLs. Endocytosis, Fc gamma R-mediated phagocytosis, and actin rearrangement were mainly affected by storage time, while cold storage alters SNARE interactions in vesicular transport and vasopressin-regulated water reabsorption [190].

A recently suggested alternative to cold storage is antipathogen treatment of platelet concentrates with Mirasol^®^ in combination with UV light irradiation was also shown to induce the PLSs and change the platelet biology. Although the technique aims to target nucleic acid, the platelet proteome and protein oxidation were affected [191,192]. The proteomics of platelets, as well as platelet-derived extracellular vesicles upon storage and treatment with Mirasol^®^, revealed induction of platelet activation, visible by affected basal platelet degranulation, upregulation of platelet factor 4, and chemokines. The elevated activation state in platelet concentrates treated with Mirasol^®^ was evident already after four days of storage [193,194]. Additionally, agitation, platelet storage containers, and solutions, alkaline conditions (pH ≥ 7.4) could influence platelet biology and cause adverse reactions in transfusions.

In the coming era of precision medicine, platelet concentrates for personalized transfusions could become a reality. The biomarkers of platelets PSL could be monitored by targeted MS integrated into a platelet transfusion medicine routine.

## 5. Future Perspective: Platelet Proteomics in Precision Medicine

Cardiovascular thromboembolic disease such as myocardial infarction, ischemic stroke, and pulmonary embolism have remained a major cause of dead and disability in Europe. Thus, it constitutes a priority to improve diagnostics and therapy in a personalized medicine setting. While classical platelet-activation markers are sensitive to pick up excessive or defective activation state, e.g., cell-surface P-selectin as a biomarker for α-granule release currently available markers have limited disease specificity. In contrast recent analyses of platelet mRNAs, microRNAs (miRNAs), and phosphoproteomics signatures show increased disease-specificity. Interestingly, mRNAs and miRNAs derived from platelets and ribosomes from are useful in assessing myeloproliferative diseases [55]. Recently the authors have shown that targeted platelet proteomics allows for quantifying cardiovascular disease biomarkers in human platelets [105] based on comparisons of a subset of peptides and corresponding proteins in diseased versus human platelets. It will be of increasing importance to assess the individual variability in the proteome of healthy subjects and patients in a personalized and precision medicine setting. Bioinformatics-based integration of “omics”, biochemical, and functional data will provide detailed information for development of novel diagnostic and pharmacologic targets that might improve patient care in precision medicine.

## 6. Conclusions

In the past years, it has become evident that platelet proteomics can provide novel insights into basic research questions and, thus, improve our understanding about the fundamental processes that regulate platelets, and contribute to treatment of platelet dysfunction. Quantitative proteomics studies will deliver detailed information about protein distributions in healthy volunteers and patients. Furthermore, the phosphorylation patterns of platelets will be useful to understand platelet activation and investigate novel therapeutic interventions. In particular, quantitative phosphoproteomic studies will pave the way for a better understanding of platelet signaling beyond the classical description of linear pathways. Although these novel technological approaches have made and will make important discoveries, the past decade showed that signaling is much more dynamic than classical approaches predicted. As future direction, unsupervised elucidation of signaling in platelets using artificial intelligence may become more important to detect so far unknown between posttranslational modifications. The vast amount of data that are produced by quantitative proteomics studies require novel data analyses strategies to integrate classical biochemical knowledge and unexpected insights from big data approaches.

This will lead to the molecular description of inhibitory and activation pathways, which are known to be modulated in disease. Given the function of platelets to facilitate thrombosis and hemostasis, quantitative (phospho)proteomic analyses will lead to molecular markers for diagnosis and prognosis of platelet dysfunction.

## Data Availability

Not applicable.

## Abbreviations

PTMs	Post-translational modifications
OCS	open canalicular system
DTS	dense tubular system
TLRs	toll-like receptors
PDI	protein disulfide isomerase
vWF	von Willebrand factor
TxA2	thromboxane A2
FcRγ	Fc receptor γ
ITAM	tyrosine-based activation motif
PLC	phospholipase C
IP3	inositol 1,4,5,-triphosphate
PRRs	pattern recognition receptors
NLRs	nod-like receptors
CLRs	C-type lectin receptors
DAMPs	damage-associated molecular patterns
MALDI	matrix-assisted laser desorption/ionization
ESI	electrospray ionization
HPLC	high-performance liquid chromatography
MS	mass spectrometry
DDA	data-dependent acquisition
DIA	data-independent acquisition
MRM	multiple reaction monitoring
PRM	parallel reaction monitoring
SRM	selected reaction monitoring
TMT	tandem mass tags
iTRAQ	isobaric tags for relative and absolute quantitation
XIC	extracted ion chromatogram
TIMS	trapped ion mobility spectrometry coupled to time-of-flight
TOF	time-of-flight mass analyzer
PASEF	parallel accumulation–aerial fragmentation
IMAC	immobilized metal ion affinity chromatography
ERLIC	electrostatic repulsion–hydrophilic interaction chromatography
SCX	strong cation exchange chromatography
SAX	strong anion exchange chromatography
CRP	collagen-related-peptide
GAPS	GTPase activating proteins
ChaFRADIC	charge-based fractional diagonal chromatography

## References

[1] Humphrey J.H. (1955). Origin of Blood Platelets. Nat. Cell Biol..

[2] Lefrançais E., Ortiz-Muñoz G., Caudrillier A., Mallavia B., Liu F., Sayah D.M., Thornton E.E., Headley M.B., David T., Coughlin T.D.S.R. (2017). The lung is a site of platelet biogenesis and a reservoir for haematopoietic progenitors. Nat. Cell Biol..

[3] Lefrançais E., Looney M.R. (2019). Platelet Biogenesis in the Lung Circulation. Physiology.

[4] Kaushansky K. (2006). Lineage-Specific Hematopoietic Growth Factors. N. Engl. J. Med..

[5] de Sauvage F.J., Hass P.E., Spencer S.D., Malloy B.E., Gurney A.L., Spencer S.A., Darbonne W.C., Henzel W.J., Wong S.C., Kuang W.-J. (1994). Stimulation of megakaryocytopoiesis and thrombopoiesis by the c-Mpl ligand. Nature.

[6] Bhat F.A., Advani J., Khan A.A., Mohan S., Pal A., Gowda H., Chakrabarti P., Prasad T.S.K., Chatterjee A. (2018). A network map of thrombopoietin signaling. J. Cell Commun. Signal..

[7] Nakamura-Ishizu A., Matsumura T., Stumpf P.S., Umemoto T., Takizawa H., Takihara Y., O’Neil A., Majeed A.B.B.A., MacArthur B.D., Suda T. (2018). Thrombopoietin Metabolically Primes Hematopoietic Stem Cells to Megakaryocyte-Lineage Differentiation. Cell Rep..

[8] Pagel O., Walter E., Jurk K., Zahedi R.P. (2016). Taking the stock of granule cargo: Platelet releasate proteomics. Platelets.

[9] Heijnen H., Van Der Sluijs P. (2015). Platelet secretory behaviour: As diverse as the granules … or not?. J. Thromb. Haemost..

[10] Escolar G., White J.G. (1991). The platelet open canalicular system: A final common pathway. Blood Cells.

[11] Heijnen H.F.G., Korporaal S.J.A., Gresele P. (2017). Platelet Morphology and Ultrastructure. Platelets in Thrombotic and Non-Thrombotic Disorders: Pathophysiology, Pharmacology and Therapeutics: An Update.

[12] Gerrard J.M., White J.G., Peterson D.A. (1978). The platelet dense tubular system: Its relationship to prostaglandin synthesis and calcium flux. Thromb. Haemost..

[13] Thon J.N., Peters C.G., Machlus K.R., Aslam R., Rowley J., MacLeod H., Devine M.T., Fuchs T.A., Weyrich A.S., Semple J.W. (2012). T granules in human platelets function in TLR9 organization and signaling. J. Cell Biol..

[14] Michelson A.D. (2013). Platelets.

[15] Ghoshal K., Bhattacharyya M. (2014). Overview of Platelet Physiology: Its Hemostatic and Nonhemostatic Role in Disease Pathogenesis. Sci. World J..

[16] Nuyttens B.P., Thijs T., Deckmyn H., Broos K. (2011). Platelet adhesion to collagen. Thromb. Res..

[17] Toonstra C., Hu Y., Zhang H. (2019). Deciphering the Roles of N-Glycans on Collagen–Platelet Interactions. J. Proteome Res..

[18] Tomaiuolo M., Matzko C.N., Poventud-Fuentes I., Weisel J.W., Brass L.F., Stalker T.J. (2019). Interrelationships between structure and function during the hemostatic response to injury. Proc. Natl. Acad. Sci. USA.

[19] Gkaliagkousi E., Passacquale G., Douma S., Zamboulis C., Ferro A. (2010). Platelet Activation in Essential Hypertension: Implications for Antiplatelet Treatment. Am. J. Hypertens..

[20] Sang Y., Roest M., de Laat B., de Groot P.G., Huskens D. (2021). Interplay between platelets and coagulation. Blood Rev..

[21] Schmaier A.A., Zou Z., Kazlauskas A., Emert-Sedlak L., Fong K.P., Neeves K.B., Maloney S.F., Diamond S.L., Kunapuli S.P., Ware J. (2009). Molecular priming of Lyn by GPVI enables an immune receptor to adopt a hemostatic role. Proc. Natl. Acad. Sci. USA.

[22] Heemskerk J.W., Harper M.T., Cosemans J.M., Poole A.W. (2011). Unravelling the different functions of protein kinase C isoforms in platelets. FEBS Lett..

[23] Crittenden J.R., Bergmeier W., Zhang Y., Piffath C.L., Liang Y., Wagner D.D., Housman D.E., Graybiel A.M. (2004). CalDAG-GEFI integrates signaling for platelet aggregation and thrombus formation. Nat. Med..

[24] Durrant T.N., van den Bosch M.T., Hers I. (2017). Integrin alphaIIbbeta3 outside-in signaling. Blood.

[25] Koupenova M., Clancy L., Corkrey H.A., Freedman J.E. (2018). Circulating Platelets as Mediators of Immunity, Inflammation, and Thrombosis. Circ. Res..

[26] Ribeiro L.S., Branco L.M., Franklin B.S. (2019). Regulation of Innate Immune Responses by Platelets. Front. Immunol..

[27] Chen Y., Zhong H., Zhao Y., Luo X., Gao W. (2020). Role of platelet biomarkers in inflammatory response. Biomark. Res..

[28] Li C., Li J., Ni H. (2020). Crosstalk between Platelets and Microbial Pathogens. Front. Immunol..

[29] Oyarzún C.P.M., Glembotsky A.C., Goette N.P., Lev P.R., De Luca G., Pietto M.C.B., Moiraghi B., Ríos M.A.C., Vicente A., Marta R.F. (2020). Platelet Toll-Like Receptors Mediate Thromboinflammatory Responses in Patients With Essential Thrombocythemia. Front. Immunol..

[30] Hottz E.D., Bozza F.A., Bozza P.T. (2018). Platelets in Immune Response to Virus and Immunopathology of Viral Infections. Front. Med..

[31] Gomez-Casado C., Villaseñor A., Rodriguez-Nogales A., Bueno J.L., Barber D., Escribese M.M. (2019). Understanding Platelets in Infectious and Allergic Lung Diseases. Int. J. Mol. Sci..

[32] Cognasse F., Hamzeh H., Chavarin P., Acquart S., Genin C., Garraud O. (2005). Evidence of Toll-like receptor molecules on human platelets. Immunol. Cell Biol..

[33] Hally K., Fauteux-Daniel S., Hamzeh-Cognasse H., Larsen P., Cognasse F. (2020). Revisiting Platelets and Toll-Like Receptors (TLRs): At the Interface of Vascular Immunity and Thrombosis. Int. J. Mol. Sci..

[34] Marcoux G., Magron A., Sut C., Laroche A., Laradi S., Hamzeh-Cognasse H., Allaeys I., Cabon O., Julien A., Garraud O. (2019). Platelet-derived extracellular vesicles convey mitochondrial DAMPs in platelet concentrates and their levels are associated with adverse reactions. Transfusion.

[35] Blair P., Rex S., Vitseva O., Beaulieu L., Tanriverdi K., Chakrabarti S., Hayashi C., Genco C.A., Iafrati M., Freedman J.E. (2009). Stimulation of Toll-Like Receptor 2 in Human Platelets Induces a Thromboinflammatory Response Through Activation of Phosphoinositide 3-Kinase. Circ. Res..

[36] Zhang G., Han J., Welch E.J., Ye R.D., Voyno-Yasenetskaya T.A., Malik A.B., Du X., Li Z. (2009). Lipopolysaccharide Stimulates Platelet Secretion and Potentiates Platelet Aggregation via TLR4/MyD88 and the cGMP-Dependent Protein Kinase Pathway. J. Immunol..

[37] Martyanov A.A., Maiorov A.S., Filkova A.A., Ryabykh A.A., Svidelskaya G.S., Artemenko E.O., Gambaryan S.P., Panteleev M.A., Sveshnikova A.N. (2020). Effects of bacterial lipopolysaccharides on platelet function: Inhibition of weak platelet activation. Sci. Rep..

[38] Clark S.R., Ma A.C., Tavener S.A., McDonald B., Goodarzi Z., Kelly M.M., Patel K.D., Chakrabarti S., McAvoy E., Sinclair G.D. (2007). Platelet TLR4 activates neutrophil extracellular traps to ensnare bacteria in septic blood. Nat. Med..

[39] Andonegui G., Kerfoot S.M., McNagny K., Ebbert K.V.J., Patel K.D., Kubes P. (2005). Platelets express functional Toll-like receptor. Blood.

[40] Anabel A.-S., Eduardo P.-C., Antonio H.-C.P., Carlos S.-M., Juana N.-M., Honorio T.-A., Nicolás V.-S., Roberto A.-R.S. (2014). Human platelets express Toll-like receptor 3 and respond to poly I:C. Hum. Immunol..

[41] Avcilar H., Eken A. (2020). Could imiquimod (Aldara 5% cream) or other TLR7 agonists be used in the treatment of COVID-19?. Med. Hypotheses.

[42] Merad M., Martin J.C. (2020). Pathological inflammation in patients with COVID-19: A key role for monocytes and macrophages. Nat. Rev. Immunol..

[43] Lund J.M., Alexopoulou L., Sato A., Karow M., Adams N.C., Gale N.W., Iwasaki A., Flavell R.A. (2004). Recognition of single-stranded RNA viruses by Toll-like receptor 7. Proc. Natl. Acad. Sci. USA.

[44] Koupenova M., Corkrey H.A., Vitseva O., Manni G., Pang C.J., Clancy L., Yao C., Rade J., Levy D., Wang J.P. (2019). The role of platelets in mediating a response to human influenza infection. Nat. Commun..

[45] Koupenova M. (2020). Potential role of platelets in COVID-19: Implications for thrombosis. Res. Pract. Thromb. Haemost..

[46] Zeiler M., Moser M., Mann M. (2014). Copy Number Analysis of the Murine Platelet Proteome Spanning the Complete Abundance Range. Mol. Cell. Proteom..

[47] Burkhart J.M., Vaudel M., Gambaryan S., Radau S., Walter U., Martens L., Geiger J., Sickmann A., Zahedi R.P. (2012). The first comprehensive and quantitative analysis of human platelet protein composition allows the comparative analysis of structural and functional pathways. Blood.

[48] Simon A.Y., Sutherland M.R., Pryzdial E.L.G. (2015). Dengue virus binding and replication by platelets. Blood.

[49] De Almeida A.J., Campos-de-Magalhães M., Brandao-Mello C.E., de Oliveira R.V., do Espirito-Santo M.P., Yoshida C.F., Lampe E. (2009). Detection of hepatitis C virus in platelets: Evaluating its relationship to antiviral therapy outcome. Hepatogastroenterology.

[50] Zucker-Franklin D., Seremetis S., Zheng Z.Y. (1990). Internalization of human immunodeficiency virus type I and other retroviruses by megakaryocytes and platelets. Blood.

[51] Hoylaerts M.F., Vanassche T., Verhamme P. (2018). Bacterial killing by platelets: Making sense of (H)IT. J. Thromb. Haemost..

[52] Krauel K., Weber C., Brandt S., Zähringer U., Mamat U., Greinacher A., Hammerschmidt S. (2012). Platelet factor 4 binding to lipid A of Gram-negative bacteria exposes PF4/heparin-like epitopes. Blood.

[53] Sung P.-S., Huang T.-F., Hsieh S.-L. (2019). Extracellular vesicles from CLEC2-activated platelets enhance dengue virus-induced lethality via CLEC5A/TLR2. Nat. Commun..

[54] Mailer R.K., Allende M., Heestermans M., Schweizer M., Deppermann C., Frye M., Pula G., Odeberg J., Gelderblom M., Rose-John S. (2021). Xenotropic and polytropic retrovirus receptor 1 regulates procoagulant platelet polyphosphate. Blood.

[55] Xu X., Gnatenko D.V., Ju J., Hitchcock I.S., Martin D.W., Zhu W., Bahou W.F. (2012). Systematic analysis of microRNA fingerprints in thrombocythemic platelets using integrated platforms. Blood.

[56] Zhang S., Liu Y., Wang X., Yang L., Li H., Wang Y., Liu M., Zhao X., Xie Y., Yang Y. (2020). SARS-CoV-2 binds platelet ACE2 to enhance thrombosis in COVID-19. J. Hematol. Oncol..

[57] Manne B.K., Denorme F., Middleton E.A., Portier I., Rowley J.W., Stubben C.J., Petrey A.C., Tolley N.D., Guo L., Cody M.J. (2020). Platelet gene expression and function in patients with COVID-19. Blood.

[58] McFadyen J.D., Stevens H., Peter K. (2020). The Emerging Threat of (Micro)Thrombosis in COVID-19 and Its Therapeutic Implications. Circ. Res..

[59] Chapman L.M., Aggrey A.A., Field D.J., Srivastava K., Ture S., Yui K., Topham D.J., Baldwin W.M., Morrell C.N. (2012). Platelets Present Antigen in the Context of MHC Class I. J. Immunol..

[60] Green S.A., Smith M., Hasley R.B., Stephany D., Harned A., Nagashima K., Abdullah S., Pittaluga S., Imamichi T., Qin J. (2015). Activated platelet-T-cell conjugates in peripheral blood of patients with HIV infection: Coupling coagulation/inflammation and T cells. AIDS.

[61] Trugilho M.R.D.O., Hottz E.D., Brunoro G.V.F., Teixeira-Ferreira A., Carvalho P.C., Salazar G.A., Zimmerman G.A., Bozza F.A., Bozza P.T., Perales J. (2017). Platelet proteome reveals novel pathways of platelet activation and platelet-mediated immunoregulation in dengue. PLoS Pathog..

[62] Qiu J., Ma J., Zhang S., Han J., Liu S. (2020). Promoting platelets is a therapeutic option to combat severe viral infection of the lung. Blood Adv..

[63] Zufferey A., Speck E.R., Machlus K.R., Aslam R., Guo L., McVey M.J., Kim M., Kapur R., Boilard E., Italiano J.E. (2017). Mature murine megakaryocytes present antigen-MHC class I molecules to T cells and transfer them to platelets. Blood Adv..

[64] Colberg L., Cammann C., Greinacher A., Seifert U. (2020). Structure and function of the ubiquitin-proteasome system in platelets. J. Thromb. Haemost..

[65] Angénieux C., Dupuis A., Gachet C., de la Salle H., Maître B. (2019). Cell surface expression of HLA I molecules as a marker of young platelets. J. Thromb. Haemost..

[66] Klose J. (1975). Protein mapping by combined isoelectric focusing and electrophoresis of mouse tissues. A novel approach to testing for induced point mutations in mammals. Humangenetik.

[67] O’Farrell P.H. (1975). High resolution two-dimensional electrophoresis of proteins. J. Biol. Chem..

[68] Michel P.E., Reymond F., Arnaud I.L., Josserand J., Girault H.H., Rossier J.S. (2003). Protein fractionation in a multicompartment device using Off-Gel™ isoelectric focusing. Electrophoresis.

[69] Gevaert K., Eggermont L., Demol H., Vandekerckhove J. (2000). A fast and convenient MALDI-MS based proteomic approach: Identification of components scaffolded by the actin cytoskeleton of activated human thrombocytes. J. Biotechnol..

[70] Marcus K., Immler D., Sternberger J., Meyer H.E. (2000). Identification of platelet proteins separated by two-dimensional gel electrophoresis and analyzed by matrix assisted laser desorption/ionization-time of flight-mass spectrometry and detection of tyrosine-phosphorylated proteins. Electrophoresis.

[71] Coppinger J.A., Cagney G., Toomey S., Kislinger T., Belton O., McRedmond J.P., Cahill D.J., Emili A., Fitzgerald D.J., Maguire P.B. (2004). Characterization of the proteins released from activated platelets leads to localization of novel platelet proteins in human atherosclerotic lesions. Blood.

[72] Garcia B.A., Smalley D.M., Cho H., Shabanowitz J., Ley K., Hunt D.F. (2005). The Platelet Microparticle Proteome. J. Proteome Res..

[73] Zahedi R.P., Lewandrowski U., Wiesner J., Wortelkamp S., Moebius J., Schütz C., Walter U., Gambaryan S., Sickmann A. (2008). Phosphoproteome of Resting Human Platelets. J. Proteome Res..

[74] Winkler W., Zellner M., Diestinger M., Babeluk R., Marchetti M., Goll A., Zehetmayer S., Bauer P., Rappold E., Miller I. (2008). Biological Variation of the Platelet Proteome in the Elderly Population and Its Implication for Biomarker Research. Mol. Cell. Proteom..

[75] García A., Prabhakar S., Brock C.J., Pearce A.C., Dwek R.A., Watson S.P., Hebestreit H.F., Zitzmann N. (2004). Extensive analysis of the human platelet proteome by two-dimensional gel electrophoresis and mass spectrometry. Proteomics.

[76] Arias-Salgado E.G., Larrucea S., Butta N., Fernández D., García-Muñoz S., Parrilla R., Ayuso M.S. (2008). Variations in platelet protein associated with arterial thrombosis. Thromb. Res..

[77] Zufferey A., Fontana P., Reny J.-L., Nolli S., Sanchez J.-C. (2012). Platelet proteomics. Mass Spectrom. Rev..

[78] Gianazza E., Brioschi M., Baetta R., Mallia A., Banfi C., Tremoli E. (2020). Platelets in Healthy and Disease States: From Biomarkers Discovery to Drug Targets Identification by Proteomics. Int. J. Mol. Sci..

[79] Vélez P., García Á. (2015). Platelet proteomics in cardiovascular diseases. Transl. Proteom..

[80] Burkhart J.M., Gambaryan S., Watson S.P., Jurk K., Walter U., Sickmann A., Heemskerk J.W.M., Zahedi R.P. (2014). What Can Proteomics Tell Us About Platelets?. Circ. Res..

[81] Hillenkamp F., Karas M., Beavis R.C., Chait B.T. (1991). Matrix-assisted laser desorption/ionization mass spectrometry of biopolymers. Anal. Chem..

[82] Fenn J.B., Mann M., Meng C.K., Wong S.F., Whitehouse C.M. (1989). Electrospray ionization for mass spectrometry of large biomolecules. Science.

[83] Marx V. (2013). Targeted proteomics. Nat. Methods.

[84] Steen H., Mann M. (2004). The ABC’s (and XYZ’s) of peptide sequencing. Nat. Rev. Mol. Cell Biol..

[85] Lee H., Chae S., Park J., Bae J., Go E.-B., Kim S.-J., Kim H., Hwang D., Lee S.-W., Lee S.-Y. (2016). Comprehensive Proteome Profiling of Platelet Identified a Protein Profile Predictive of Responses to An Antiplatelet Agent Sarpogrelate. Mol. Cell. Proteom..

[86] Thompson A., Schäfer J., Kuhn K., Kienle S., Schwarz J., Schmidt G., Neumann T., Hamon C. (2003). Tandem mass tags: A novel quantification strategy for comparative analysis of complex protein mixtures by MS/MS. Anal. Chem..

[87] Ross P.L., Huang Y.N., Marchese J.N., Williamson B., Parker K., Hattan S., Khainovski N., Pillai S., Dey S., Daniels S. (2004). Multiplexed Protein Quantitation in Saccharomyces cerevisiae Using Amine-reactive Isobaric Tagging Reagents. Mol. Cell. Proteom..

[88] Beck F., Burkhart J.M., Geiger J., Zahedi R.P., Sickmann A. (2012). Robust workflow for iTRAQ-based peptide and protein quantification. Methods Mol. Biol..

[89] Bąchor R., Waliczek M., Stefanowicz P., Szewczuk Z. (2019). Trends in the Design of New Isobaric Labeling Reagents for Quantitative Proteomics. Molecules.

[90] Beck F., Geiger J., Gambaryan S., Solari F.A., Dell’Aica M., Loroch S., Mattheij N.J., Mindukshev I., Pötz O., Jurk K. (2017). Temporal quantitative phosphoproteomics of ADP stimulation reveals novel central nodes in platelet activation and inhibition. Blood.

[91] Schweigel H., Geiger J., Beck F., Buhs S., Gerull H., Walter U., Sickmann A., Nollau P., Geiger J. (2013). Deciphering of ADP-induced, phosphotyrosine-dependent signaling networks in human platelets by Src-homology 2 region (SH2)-profiling. Proteomics.

[92] Beck F., Geiger J., Gambaryan S., Veit J., Vaudel M., Nollau P., Kohlbacher O., Martens L., Walter U., Sickmann A. (2014). Time-resolved characterization of cAMP/PKA-dependent signaling reveals that platelet inhibition is a concerted process involving multiple signaling pathways. Blood.

[93] Solari F.A., Mattheij N.J., Burkhart J.M., Swieringa F., Collins P.W., Cosemans J.M., Sickmann A., Heemskerk J.W., Zahedi R.P. (2016). Combined Quantification of the Global Proteome, Phosphoproteome, and Proteolytic Cleavage to Characterize Altered Platelet Functions in the Human Scott Syndrome. Mol. Cell. Proteom..

[94] Sims M.C., Mayer L., Collins J.H., Bariana T.K., Megy K., Lavenu-Bombled C., Seyres D., Kollipara L., Burden F.S., Greene D. (2020). Novel manifestations of immune dysregulation and granule defects in gray platelet syndrome. Blood.

[95] Swieringa F., Solari F.A., Pagel O., Beck F., Huang J., Feijge M.A.H., Jurk K., Körver-Keularts I.M.L.W., Mattheij N.J.A., Faber J. (2020). Impaired iloprost-induced platelet inhibition and phosphoproteome changes in patients with confirmed pseudohypoparathyroidism type Ia, linked to genetic mutations in GNAS. Sci. Rep..

[96] Loroch S., Trabold K., Gambaryan S., Reiß C., Schwierczek K., Fleming I., Sickmann A., Behnisch W., Zieger B., Zahedi R.P. (2017). Alterations of the platelet proteome in type I Glanzmann thrombasthenia caused by different homozygous delG frameshift mutations in ITGA2B. Thromb. Haemost..

[97] Michalski A., Cox J., Mann M. (2011). More than 100,000 Detectable Peptide Species Elute in Single Shotgun Proteomics Runs but the Majority is Inaccessible to Data-Dependent LC−MS/MS. J. Proteome Res..

[98] Bekker-Jensen D.B., Bernhardt O.M., Hogrebe A., Martinez-Val A., Verbeke L., Gandhi T., Kelstrup C.D., Reiter L., Olsen J.V. (2020). Rapid and site-specific deep phosphoproteome profiling by data-independent acquisition without the need for spectral libraries. Nat. Commun..

[99] Mun D.-G., Renuse S., Saraswat M., Madugundu A., Udainiya S., Kim H., Park S.-K.R., Zhao H., Nirujogi R.S., Na C.H. (2020). PASS-DIA: A Data-Independent Acquisition Approach for Discovery Studies. Anal. Chem..

[100] Gillet L.C., Navarro P., Tate S., Röst H., Selevsek N., Reiter L., Bonner R., Aebersold R. (2012). Targeted Data Extraction of the MS/MS Spectra Generated by Data-independent Acquisition: A New Concept for Consistent and Accurate Proteome Analysis. Mol. Cell. Proteom..

[101] Ludwig C., Gillet L., Rosenberger G., Amon S., Collins B.C., Aebersold R. (2018). Data-independent acquisition-based SWATH-MS for quantitative proteomics: A tutorial. Mol. Syst. Biol..

[102] Domon B., Aebersold R. (2010). Options and considerations when selecting a quantitative proteomics strategy. Nat. Biotechnol..

[103] Zhang C.-C., Li R., Jiang H., Lin S., Rogalski J.C., Liu K., Kast J. (2014). Development and Application of a Quantitative Multiplexed Small GTPase Activity Assay Using Targeted Proteomics. J. Proteome Res..

[104] Liu S.-Y., Yuan D., Sun R.-J., Zhu J.-J., Shan N.-N. (2020). Significant reductions in apoptosis-related proteins (HSPA6, HSPA8, ITGB3, YWHAH, and PRDX6) are involved in immune thrombocytopenia. J. Thromb. Thrombolysis.

[105] Malchow S., Loosse C., Sickmann A., Lorenz C. (2017). Quantification of Cardiovascular Disease Biomarkers in Human Platelets by Targeted Mass Spectrometry. Proteomes.

[106] Guo L., Wang Q., Weng L., Hauser L.A., Strawser C.J., Rocha A.G., Dancis A., Mesaros C.A., Lynch D.R., Blair I.A. (2018). Liquid Chromatography-High Resolution Mass Spectrometry Analysis of Platelet Frataxin as a Protein Biomarker for the Rare Disease Friedreich’s Ataxia. Anal. Chem..

[107] Meier F., Beck S., Grassl N., Lubeck M., Park M.A., Raether O., Mann M. (2015). Parallel Accumulation–Serial Fragmentation (PASEF): Multiplying Sequencing Speed and Sensitivity by Synchronized Scans in a Trapped Ion Mobility Device. J. Proteome Res..

[108] Meier F., Brunner A.-D., Koch S., Koch H., Lubeck M., Krause M., Goedecke N., Decker J., Kosinski T., Park M.A. (2018). Online Parallel Accumulation–Serial Fragmentation (PASEF) with a Novel Trapped Ion Mobility Mass Spectrometer. Mol. Cell. Proteom..

[109] Budnik B., Levy E., Harmange G., Slavov N. (2018). SCoPE-MS: Mass spectrometry of single mammalian cells quantifies proteome heterogeneity during cell differentiation. Genome Biol..

[110] Chen A.T., Franks A., Slavov N. (2019). DART-ID increases single-cell proteome coverage. PLoS Comput. Biol..

[111] Levy E., Slavov N. (2018). Single cell protein analysis for systems biology. Essays Biochem..

[112] Marx R.E. (2001). Platelet-Rich Plasma (PRP): What Is PRP and What Is Not PRP?. Implant. Dent..

[113] Greening D.W., Sparrow R.L., Simpson R.J. (2011). Preparation of platelet concentrates. Methods Mol. Biol..

[114] Wrzyszcz A., Urbaniak J., Sapa A., Woźniak M. (2016). An efficient method for isolation of representative and contamination-free population of blood platelets for proteomic studies. Platelets.

[115] Trichler S.A., Bulla S.C., Thomason J., Lunsford K.V., Bulla C. (2013). Ultra-pure platelet isolation from canine whole blood. BMC Vet. Res..

[116] Kim C.-J., Ki D.Y., Park J., Sunkara V., Kim T.-H., Min Y., Cho Y.-K. (2020). Fully automated platelet isolation on a centrifugal microfluidic device for molecular diagnostics. Lab Chip.

[117] Burkhart J.M., Schumbrutzki C., Wortelkamp S., Sickmann A., Zahedi R.P. (2012). Systematic and quantitative comparison of digest efficiency and specificity reveals the impact of trypsin quality on MS-based proteomics. J. Proteom..

[118] Maia T.M., Staes A., Plasman K., Pauwels J., Boucher K., Argentini A., Martens L., Montoye T., Gevaert K., Impens F. (2020). Simple Peptide Quantification Approach for MS-Based Proteomics Quality Control. ACS Omega.

[119] Fu Q., Johnson C.W., Wijayawardena B.K., Kowalski M.P., Kheradmand M., Van Eyk J.E. (2020). A Plasma Sample Preparation for Mass Spectrometry using an Automated Workstation. J. Vis. Exp..

[120] Chiva C., Olivella R., Borràs E., Espadas G., Pastor O., Solé A., Sabido E. (2018). QCloud: A cloud-based quality control system for mass spectrometry-based proteomics laboratories. PLoS ONE.

[121] Kim T., Chen I.R., Parker B.L., Humphrey S.J., Crossett B., Cordwell S.J., Yang P., Yang J.Y.H. (2019). QCMAP: An Interactive Web-Tool for Performance Diagnosis and Prediction of LC-MS Systems. Proteomics.

[122] Van Houtven J., Agten A., Boonen K., Baggerman G., Hooyberghs J., Laukens K., Valkenborg D., Hooybergs J. (2019). QCQuan: A Web Tool for the Automated Assessment of Protein Expression and Data Quality of Labeled Mass Spectrometry Experiments. J. Proteome Res..

[123] Mnatsakanyan R., Shema G., Basik M., Batist G., Borchers C.H., Sickmann A., Zahedi R.P. (2018). Detecting post-translational modification signatures as potential biomarkers in clinical mass spectrometry. Expert Rev. Proteom..

[124] Sharma S., Toledo O., Hedden M., Lyon K.F., Brooks S.B., David R.P., Limtong J., Newsome J.M., Novakovic N., Rajasekaran S. (2016). The Functional Human C-Terminome. PLoS ONE.

[125] Sharma S., Schiller M.R. (2019). The carboxy-terminus, a key regulator of protein function. Crit. Rev. Biochem. Mol. Biol..

[126] Looße C., Swieringa F., Heemskerk J.W., Sickmann A., Lorenz C. (2018). Platelet proteomics: From discovery to diagnosis. Expert Rev. Proteom..

[127] Virág D., Dalmadi-Kiss B., Vékey K., Drahos L., Klebovich I., Antal I., Ludányi K. (2019). Current Trends in the Analysis of Post-translational Modifications. Chromatographia.

[128] Dickhut C., Radau S., Zahedi R.P. (2014). Fast, efficient, and quality-controlled phosphopeptide enrichment from minute sample amounts using titanium dioxide. Methods Mol. Biol..

[129] Fíla J., Honys D. (2011). Enrichment techniques employed in phosphoproteomics. Amino Acids.

[130] Loroch S., Schommartz T., Brune W., Zahedi R.P., Sickmann A. (2015). Multidimensional electrostatic repulsion–hydrophilic interaction chromatography (ERLIC) for quantitative analysis of the proteome and phosphoproteome in clinical and biomedical research. Biochim. Biophys. Acta (BBA) Proteins Proteom..

[131] Pagel O., Loroch S., Sickmann A., Zahedi R.P. (2015). Current strategies and findings in clinically relevant post-translational modification-specific proteomics. Expert Rev. Proteom..

[132] Stolze S.C., Nakagami H. (2020). Targeted Quantification of Phosphopeptides by Parallel Reaction Monitoring (PRM). Methods Mol. Biol..

[133] Maguire P.B., Wynne K.J., Harney D.F., O’Donoghue N.M., Stephens G., Fitzgerald D.J. (2002). Identification of the phosphotyrosine proteome from thrombin activated platelets. Proteomics.

[134] García Á., Senis Y.A., Antrobus R., Hughes C.E., Dwek R.A., Watson S.P., Zitzmann N. (2006). A global proteomics approach identifies novel phosphorylated signaling proteins in GPVI-activated platelets: Involvement of G6f, a novel platelet Grb2-binding membrane adapter. Proteomics.

[135] Schulz C., Leuschen N.V., Fröhlich T., Lorenz M., Pfeiler S., Gleissner C.A., Kremmer E., Kessler M., Khandoga A.G., Engelmann B. (2010). Identification of novel downstream targets of platelet glycoprotein VI activation by differential proteome analysis: Implications for thrombus formation. Blood.

[136] Bleijerveld O.B., Van Holten T.C., Preisinger C., Van Der Smagt J.J., Farndale R.W., Kleefstra T., Willemsen M.H., Urbanus R.T., De Groot P.G., Heck A.J. (2013). Targeted Phosphotyrosine Profiling of Glycoprotein VI Signaling Implicates Oligophrenin-1 in Platelet Filopodia Formation. Arter. Thromb. Vasc. Biol..

[137] García Á., Prabhakar S., Hughan S., Anderson T.W., Brock C.J., Pearce A.C., Dwek R.A., Watson S.P., Hebestreit H.F., Zitzmann N. (2004). Differential proteome analysis of TRAP-activated platelets: Involvement of DOK-2 and phosphorylation of RGS proteins. Blood.

[138] Gachet C. (2015). Antiplatelet drugs: Which targets for which treatments?. J. Thromb. Haemost..

[139] Schmidt G.J., Reumiller C.M., Ercan H., Resch U., Butt E., Heber S., Liutkevičiūte Z., Basílio J., Schmid J.A., Assinger A. (2019). Comparative proteomics reveals unexpected quantitative phosphorylation differences linked to platelet activation state. Sci. Rep..

[140] Makhoul S., Walter E., Pagel O., Walter U., Sickmann A., Gambaryan S., Smolenski A., Zahedi R.P., Jurk K. (2018). Effects of the NO/soluble guanylate cyclase/cGMP system on the functions of human platelets. Nitric Oxide.

[141] Smolenski A. (2012). Novel roles of cAMP/cGMP-dependent signaling in platelets. J. Thromb. Haemost..

[142] Gambaryan S., Tsikas D. (2015). A review and discussion of platelet nitric oxide and nitric oxide synthase: Do blood platelets produce nitric oxide from l-arginine or nitrite?. Amino Acids.

[143] Gambaryan S., Kobsar A., Hartmann S., Birschmann I., Kuhlencordt P.J., Müller-Esterl W., Lohmann S.M., Walter U. (2008). NO-synthase-NO-independent regulation of human and murine platelet soluble guanylyl cyclase activity. J. Thromb. Haemost..

[144] Subramanian H., Rukoyatkina N., Herterich S., Walter U., Gambaryan S. (2013). Soluble guanylyl cyclase is the only enzyme responsible for cyclic guanosine monophosphate synthesis in human platelets. Thromb. Haemost..

[145] El-Daher S.S., Patel Y., Siddiqua A., Hassock S., Edmunds S., Maddison B., Patel G., Goulding D., Lupu F., Wojcikiewicz R.J. (2000). Distinct localization and function of (1,4,5)IP(3) receptor subtypes and the (1,3,4,5)IP(4) receptor GAP1(IP4BP) in highly purified human platelet membranes. Blood.

[146] Antl M., von Brühl M.L., Eiglsperger C., Werner M., Konrad I., Kocher T., Wilm M., Hofmann F., Massberg S., Schlossmann J. (2007). IRAG mediates NO/cGMP-dependent inhibition of platelet aggregation and thrombus formation. Blood.

[147] Volz J., Kusch C., Beck S., Popp M., Vögtle T., Meub M., Scheller I., Heil H.S., Preu J., Schuhmann M.K. (2020). BIN2 orchestrates platelet calcium signaling in thrombosis and thrombo-inflammation. J. Clin. Investig..

[148] Aburima A., Walladbegi K., Wake J.D., Naseem K.M. (2017). cGMP signaling inhibits platelet shape change through regulation of the RhoA-Rho Kinase-MLC phosphatase signaling pathway. J. Thromb. Haemost..

[149] Aburima A., Wraith K.S., Raslan Z., Law R., Magwenzi S., Naseem K.M. (2013). cAMP signaling regulates platelet myosin light chain (MLC) phosphorylation and shape change through targeting the RhoA-Rho kinase-MLC phosphatase signaling pathway. Blood.

[150] Ellerbroek S.M., Wennerberg K., Burridge K. (2003). Serine Phosphorylation Negatively Regulates RhoA in Vivo. J. Biol. Chem..

[151] Comer S., Nagy Z., Bolado A., Von Kriegsheim A., Gambaryan S., Walter U., Pagel O., Zahedi R.P., Jurk K., Smolenski A. (2020). The RhoA regulators Myo9b and GEF-H1 are targets of cyclic nucleotide-dependent kinases in platelets. J. Thromb. Haemost..

[152] Nagy Z., Wynne K., von Kriegsheim A., Gambaryan S., Smolenski A. (2015). Cyclic Nucleotide-dependent Protein Kinases Target ARHGAP17 and ARHGEF6 Complexes in Platelets. J. Biol. Chem..

[153] Siess W., Winegar D.A., Lapetina E.G. (1990). Rap1-b is phosphorylated by protein kinase a in intact human platelets. Biochem. Biophys. Res. Commun..

[154] Hoffmeister K.M., Felbinger T.W., Falet H., Denis C.V., Bergmeier W., Mayadas T.N., Von Andrian U.H., Wagner D.D., Stossel T.P., Hartwig J.H. (2003). The Clearance Mechanism of Chilled Blood Platelets. Cell.

[155] Subramanian H., Zahedi R.P., Sickmann A., Walter U., Gambaryan S. (2013). Phosphorylation of CalDAG-GEFI by protein kinase A prevents Rap1b activation. J. Thromb. Haemost..

[156] Hoffmeister M., Riha P., Neumüller O., Danielewski O., Schultess J., Smolenski A.P. (2008). Cyclic Nucleotide-dependent Protein Kinases Inhibit Binding of 14-3-3 to the GTPase-activating Protein Rap1GAP2 in Platelets. J. Biol. Chem..

[157] Jones C.I., Barrett N.E., Moraes L.A., Gibbins J.M., Jackson D.E. (2012). Endogenous inhibitory mechanisms and the regulation of platelet function. Methods Mol. Biol..

[158] Unsworth A.J., Flora G.D., Sasikumar P., Bye A.P., Sage T., Kriek N., Crescente M., Gibbins J.M. (2017). RXR Ligands Negatively Regulate Thrombosis and Hemostasis. Arter. Thromb. Vasc. Biol..

[159] Stefanini L., Bergmeier W. (2018). Negative regulators of platelet activation and adhesion. J. Thromb. Haemost..

[160] Klockenbusch C., Walsh G.M., Brown L.M., Hoffman M.D., Ignatchenko V., Kislinger T., Kast J. (2014). Global Proteome Analysis Identifies Active Immunoproteasome Subunits in Human Platelets. Mol. Cell. Proteom..

[161] Swatek K.N., Komander D. (2016). Ubiquitin modifications. Cell Res..

[162] Peng J., Schwartz D.T., Elias J.E., Thoreen C.C., Cheng D., Marsischky G., Roelofs J., Finley D., Gygi S.P. (2003). A proteomics approach to understanding protein ubiquitination. Nat. Biotechnol..

[163] Xu G., Paige J.S., Jaffrey S.R. (2010). Global analysis of lysine ubiquitination by ubiquitin remnant immunoaffinity profiling. Nat. Biotechnol..

[164] Dangelmaier C.A., Quinter P.G., Jin J., Tsygankov A.Y., Kunapuli S.P., Daniel J.L. (2005). Rapid ubiquitination of Syk following GPVI activation in platelets. Blood.

[165] Unsworth A.J., Bombik I., Pinto-Fernandez A., McGouran J.F., Konietzny R., Zahedi R.P., Watson S.P., Kessler B.M., Pears C.J. (2018). Human Platelet Protein Ubiquitylation and Changes following GPVI Activation. Thromb. Haemost..

[166] Rogers L.D., Overall C.M. (2013). Proteolytic Post-translational Modification of Proteins: Proteomic Tools and Methodology. Mol. Cell. Proteom..

[167] Shema G., Nguyen M.T.N., Solari F.A., Loroch S., Venne A.S., Kollipara L., Sickmann A., Verhelst S.H., Zahedi R.P. (2018). Simple, scalable, and ultrasensitive tip-based identification of protease substrates. Mol. Cell. Proteom..

[168] Gutmann C., Joshi A., Mayr M. (2020). Platelet “-omics” in health and cardiovascular disease. Atherosclerosis.

[169] Parguiña A.F., Grigorian-Shamajian L., Agra R.M., Teijeira-Fernández E., Rosa I., Alonso J., Viñuela-Roldán J.E., Seoane A., González-Juanatey J.R., García A. (2010). Proteins involved in platelet signaling are differentially regulated in acute coronary syndrome: A proteomic study. PLoS ONE.

[170] Maguire P.B., Parsons M.E., Szklanna P.B., Zdanyte M., Münzer P., Chatterjee M., Wynne K., Rath D., Comer S.P., Hayden M. (2020). Comparative Platelet Releasate Proteomic Profiling of Acute Coronary Syndrome versus Stable Coronary Artery Disease. Front. Cardiovasc. Med..

[171] Sabrkhany S., Kuijpers M.J.E., Knol J.C., Olde Damink S.W.M., Dingemans A.C., Verheul H.M., Piersma S.R., Pham T.V., Griffioen A.W., Oude Egbrink M.G.A. (2018). Exploration of the platelet proteome in patients with early-stage cancer. J. Proteom..

[172] Sereni L., Castiello M.C., Marangoni F., Anselmo A., di Silvestre D., Motta S., Draghici E., Mantero S., Thrasher A.J., Giliani S. (2018). Autonomous role of Wiskott-Aldrich syndrome platelet deficiency in inducing autoimmunity and inflammation. J. Allergy Clin. Immunol..

[173] Jalal D.I., Chonchol M., Targher G. (2010). Disorders of hemostasis associated with chronic kidney disease. Semin. Thromb. Hemost..

[174] Lutz J., Menke J., Sollinger D., Schinzel S., Thürmel K. (2014). Haemostasis in chronic kidney disease. Nephrol. Dial. Transplant..

[175] Velasquez M.T., Centron P., Barrows I., Dwivedi R., Raj D.S. (2018). Gut Microbiota and Cardiovascular Uremic Toxicities. Toxins (Basel).

[176] Fryc J., Naumnik B. (2021). Thrombolome and Its Emerging Role in Chronic Kidney Diseases. Toxins (Basel).

[177] Ravid J.D., Chitalia V.V. (2020). Molecular Mechanisms Underlying the Cardiovascular Toxicity of Specific Uremic Solutes. Cells.

[178] Yang K., Du C., Wang X., Li F., Xu Y., Wang S., Chen S., Chen F., Shen M., Chen M. (2017). Indoxyl sulfate induces platelet hyperactivity and contributes to chronic kidney disease-associated thrombosis in mice. Blood.

[179] da Cunha R.S., Santos A.F., Barreto F.C., Stinghen A.E.M. (2020). How do Uremic Toxins Affect the Endothelium?. Toxins (Basel).

[180] Ng M.S.Y., Tung J.-P., Fraser J.F. (2018). Platelet Storage Lesions: What More Do We Know Now?. Transfus. Med. Rev..

[181] Shea S.M., Thomas K.A., Spinella P.C. (2019). The effect of platelet storage temperature on haemostatic, immune, and endothelial function: Potential for personalised medicine. Blood Transfus..

[182] Holme S. (1998). Storage and quality assessment of platelets. Vox Sang..

[183] Aubron C., Flint A.W.J., Ozier Y., McQuilten Z. (2018). Platelet storage duration and its clinical and transfusion outcomes: A systematic review. Crit. Care.

[184] Handigund M., Cho Y.G. (2015). Insights into Platelet Storage and the Need for Multiple Approaches. Ann. Clin. Lab. Sci..

[185] Thiele T., Steil L., Gebhard S., Scharf C., Hammer E., Brigulla M., Lubenow N., Clemetson K.J., Völker U., Greinacher A. (2007). Profiling of alterations in platelet proteins during storage of platelet concentrates. Transfusion.

[186] Rijkers M., Eshof B.L.V.D., Van Der Meer P.F., Van Alphen F.P.J., De Korte D., Leebeek F.W.G., Meijer A.B., Voorberg J., Jansen A.J.G. (2017). Monitoring storage induced changes in the platelet proteome employing label free quantitative mass spectrometry. Sci. Rep..

[187] Jansen A.J., Josefsson E.C., Rumjantseva V., Liu Q.P., Falet H., Bergmeier W., Cifuni S.M., Sackstein R., von Andrian U.H., Wagner D.D. (2012). Desialylation accelerates platelet clearance after refrigeration and initiates GPIbalpha metalloproteinase-mediated cleavage in mice. Blood.

[188] Rumjantseva V., Grewal P.K., Wandall H.H., Josefsson E.C., Sørensen A.L., Larson G., Marth J.D., Hartwig J.H., Hoffmeister K.M. (2009). Dual roles for hepatic lectin receptors in the clearance of chilled platelets. Nat. Med..

[189] Kleinveld D.J., Sloos P.H., Noorman F., Maas M.A.W., Kers J., Rijnhout T.W., Zoodsma M., Hoencamp R., Hollmann M.W., Juffermans N.P. (2020). The use of cryopreserved platelets in a trauma-induced hemorrhage model. Transfusion.

[190] Wang S., Jiang T., Fan Y., Zhao S. (2018). A proteomic approach reveals the variation in human platelet protein composition after storage at different temperatures. Platelets.

[191] Kaiser-Guignard J., Canellini G., Lion N., Abonnenc M., Osselaer J.-C., Tissot J.-D. (2014). The clinical and biological impact of new pathogen inactivation technologies on platelet concentrates. Blood Rev..

[192] Sonego G., Abonnenc M., Crettaz D., Lion N., Tissot J.-D., Prudent M. (2020). Irreversible oxidations of platelet proteins after riboflavin-UVB pathogen inactivation. Transfus. Clin. Biol..

[193] Hermida-Nogueira L., Barrachina M.N., Izquierdo I., García-Vence M., Lacerenza S., Bravo S., Castrillo A., García Á. (2020). Proteomic analysis of extracellular vesicles derived from platelet concentrates treated with Mirasol(R) identifies biomarkers of platelet storage lesion. J. Proteom..

[194] Salunkhe V., De Cuyper I.M., Papadopoulos P., Van Der Meer P.F., Daal B.B., Villa-Fajardo M., De Korte D., Berg T.K.V.D., Gutiérrez L. (2018). A comprehensive proteomics study on platelet concentrates: Platelet proteome, storage time and Mirasol pathogen reduction technology. Platelets.

